# Cannabidiol inhibits both human K_V_7.1 and K_V_7.1/KCNE1 channels through distinct sites

**DOI:** 10.1038/s41401-025-01742-0

**Published:** 2026-03-03

**Authors:** AS Kusay, M. Pökl, I. Hiniesto-Iñigo, A. Sridhar, L. Delemotte, SI Liin

**Affiliations:** 1 Department of Biomedical and Clinical Sciences, Linköping University, SE-581 85 Linköping, Sweden; 2 Department of Applied Physics, Science for Life Laboratory, KTH Royal Institute of Technology, Stockholm, Sweden

**Keywords:** electrophysiology, molecular modelling, binding site, cannabidiol, KCNQ1, KCNE1

## Abstract

Several essential physiological systems express voltage-gated potassium channels within the K_V_7 family (comprising K_V_7.1–7.5), sometimes also co-assembled with auxiliary subunits in the KCNE family (comprising KCNE1–5). An ongoing challenge to K_V_7 drug development is creating subtype-selective compounds to limit adverse effects. Prior work has shown that the antiepileptic cannabidiol (CBD), a pan-K_V_7 modulator, inhibits cardiac- and epithelia-associated K_V_7.1 and K_V_7.1/KCNE1 channels, while activating neuronal K_V_7 subtypes (K_V_7.2–7.5). However, little is known about the binding sites through which CBD mediates inhibitory effects on K_V_7.1 and K_V_7.1/KCNE1, limiting insight towards the development of selective K_V_7 modulators. To address this knowledge gap, we used a combination of the Chai-1 artificial intelligence model (to generate CBD binding site predictions in human K_V_7.1 and K_V_7.1/KCNE1 channels), site-directed mutagenesis and electrophysiology of these channels expressed in *Xenopus laevis* oocytes (to corroborate CBD binding site predictions), and molecular dynamics simulations (to study the biophysical mechanisms underlying CBD binding). We found that CBD binds to two unique sites within K_V_7.1 and K_V_7.1/KCNE1. In K_V_7.1 alone, CBD was bound to an intrasubunit S5–S6 pore domain binding site; referred to as the S5–S6 site. In K_V_7.1/KCNE1, the addition of the KCNE1 subunit created a novel binding site for CBD, sandwiched between two K_V_7.1 subunits and one KCNE1 subunit; referred to here as the S6–S5’–E1 site. Molecular dynamics simulations showed that CBD binding to the S6–S5’–E1 K_V_7.1/KCNE1 site closes off the K_V_7.1 S5–S6 site. A sequence comparison between K_V_7 channels revealed key amino acid differences at both the S5–S6 and S6–S5’–E1 sites relative to neuronal K_V_7s. These support the notion that CBD binds differently in K_V_7.1 and K_V_7.1/KCNE1 channels in accordance with its unique inhibitory pharmacological effects on these channels compared to the activating effect in neuronal K_V_7s. Thus, we provide support for K_V_7.1 and K_V_7.1/KCNE1 being inhibited by CBD via distinct binding sites, which can guide future research focused on the rational development of drugs that avoid inhibitory effects on K_V_7.1 and K_V_7.1/KCNE1 channels or utilize these sites to modulate channel activity.

## Introduction

The K_V_7.1/KCNE1 channel, generating the slow-delayed component of the outward potassium current (*I*_Ks_), plays a crucial role in cardiac repolarisation [1, 2]. Drug-induced inhibition of the K_V_7.1/KCNE1 channel may result in proarrhythmic cardiac action potential prolongation. Indeed, clinically used drugs like the anti-inflammatory celecoxib and the anti-malarial quinidine, which are known to inhibit K_V_7.1/KCNE1 channels, increase the risk of adverse cardiac events [3, 4]. In contrast, K_V_7.1/KCNE1 channel activators have been put forward as promising compounds for treating cardiac arrhythmias caused by impaired K_V_7.1/KCNE1 channel function or arrhythmias related to delayed cardiac repolarisation in general [5–7]. Besides the heart, K_V_7.1 is expressed in other tissues like epithelia, either co-assembled with other KCNE subunits or potentially as K_V_7.1 alone [8, 9]. Evidently, detailed knowledge of drug binding sites in K_V_7.1 and K_V_7.1/KCNE1 is vital to avoid adverse effects and drive rational drug development initiatives.

**Fig. 1:**
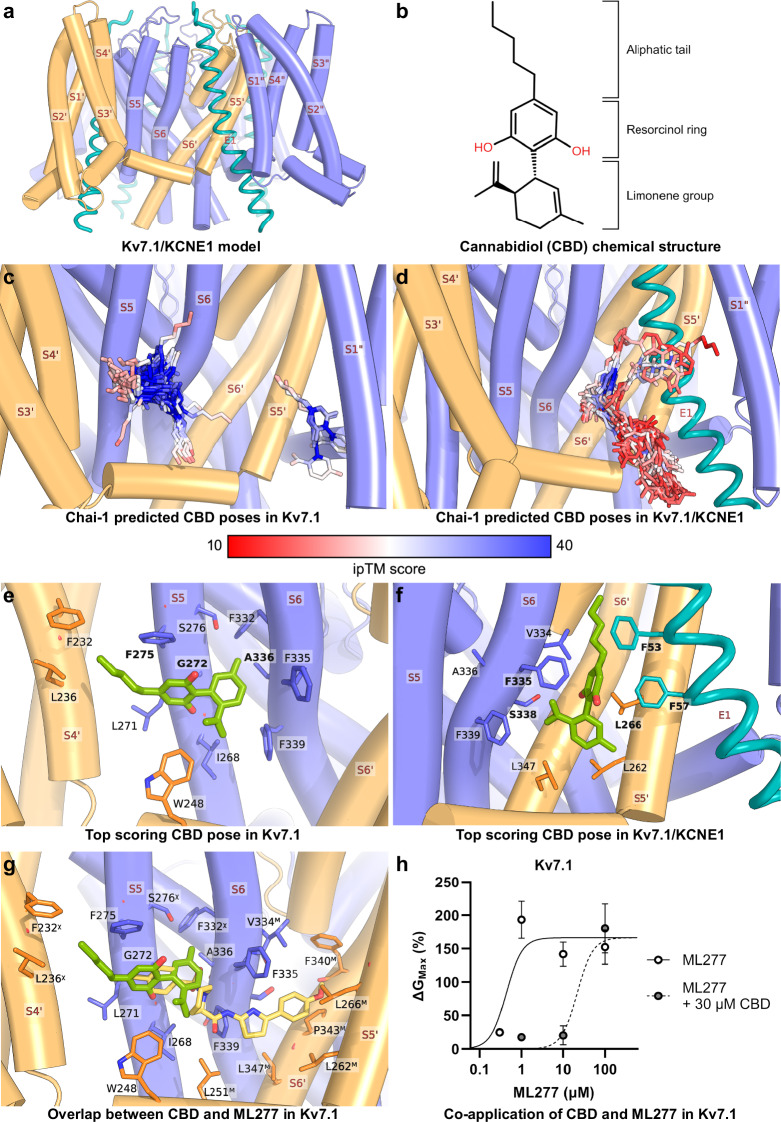
Prediction of CBD binding to human K_V_7.1 and K_V_7.1/KCNE1. **a** Topological sideview of the K_V_7.1/KCNE1 channel complex, subunits labelled with ‘ and “ suffixes indicate different K_V_7.1 subunits, the KCNE1 subunit is labelled as E1. **b** Chemical structure of cannabidiol with sub-structures highlighted. Top 50 predicted Chai-1 binding poses for CBD ranked by the ipTM score in K_V_7.1 (**c**) and in K_V_7.1/KCNE1 (**d**). The atoms of CBD are coloured in a blue-white-red spectrum representing an ipTM score range of 10 (red) to 40 (blue), this is reflected in a colour bar. Top-scoring pose for CBD in K_V_7.1 (**e**) and K_V_7.1/KCNE1 (**f**), only residues adjacent to CBD are displayed, bolded labels indicate key interacting residues. **g** Overlay between top-scoring CBD pose in K_V_7.1 and ML277 from the K_V_7.1 structure (PDB: 7XNL) [17]. Each Chai-1 prediction had 4 CBD molecules (one per PD-interface), all 4 were identical in conformation; therefore, in all panels, CBD poses from a single interface are shown. The right subunit of each interface is coloured orange, all other subunits are coloured in purple-blue, the KCNE1 subunit is coloured in teal where applicable. In **e**–**g**, CBD is coloured in green while ML277 is coloured in yellow. In (**g**), residues adjacent to CBD or ML277 are shown; a “χ” and “M” superscript is used to denote residues within 4 Å of CBD or ML277, respectively; residues within 4 Å of both do not have a superscript. **h** Comparison of the effect of ML277 alone (open circles) and ML277 in the presence of 30 µM CBD (filled circles, *Xenopus laevis* oocytes pretreated with 30 µM CBD). Data is displayed as mean ± SEM. *n* = 5–8 oocytes. Best fit for ML277 alone on K_V_7.1: EC_50_ = 0.43 µM (95% CI: 0.284–0.657, *R*^2^ = 0.501). Best fit for ML277 in the presence of CBD on K_V_7.1: EC_50_ = 21.4 µM (95% CI: 11.9–53.32, *R*^2^ = 0.678). *E*_Max_ (i.e. maximal increase in *G*_Max_ induced by ML277) was shared (+166%), and the Hill slope set to 2.3 based on previous reports [51].

The K_V_7.1/KCNE1 channel is co-assembled from the voltage-gated potassium (K_V_) channel α-subunit K_V_7.1, encoded by the *KCNQ1* gene, and the auxiliary subunit KCNE1, encoded by the *KCNE1* gene. The K_V_7.1 subunits are arranged as a tetramer in an interleaved domain-swapped conformation. This creates interfaces between several K_V_7 subunits; therefore, we label the helices from subunits 2 and 3 with the suffixes ‘ and “, respectively, to differentiate them from subunit 1 in all figures (exemplified in Fig. 1a). Each subunit contains six transmembrane helices (S1–S6), the S1–S4 helices comprise the voltage-sensing domain (VSD) while the S5–S6 helices form the ion-conducting pore domain (PD) [10]. K_V_7.1 functions as a K_V_ channel by itself, however, co-assembly with up to four KCNE1 subunits (Fig. 1a), confers biophysical properties characteristic of the native *I*_Ks_ (like slow activation kinetics and shifted voltage-dependence of channel activation) [2].

The challenge in developing drugs targeting K_V_7 channels, including K_V_7.1 and K_V_7.1/KCNE1, lies in the difficulty of making channel subtype-selective compounds. This is highlighted by the anti-epileptic drug retigabine, which was withdrawn from the market due to adverse effects, like urinary retention [11]. These adverse effects are thought to occur because retigabine binds to K_V_7.2–K_V_7.5 channels rather than being selective for the neuronal K_V_7.2/7.3 heteromeric channels [11]. The retigabine binding site has been extensively characterised, and was ultimately resolved in the K_V_7.2-bound retigabine structures [12]. Ligands at the site resolved in complex with K_V_7.2 include: retigabine, CBD, HN37, Ebio1, Ebio2 and Ebio3 [12–15]. This detailed binding site characterisation has enabled rational drug development initiatives leading to the discovery of more efficacious subtype-selective compounds than retigabine [14, 16]. In contrast, to date, K_V_7.1 structures have been resolved with only one activator, ML277 [17, 18], and there are no K_V_7.1/KCNE1 structures with ligands (although two studies with apo structures were published at the time of writing) [19, 20], further adding to the challenge of rational drug development initiatives.

A compound with notable parallels to retigabine is the phytocannabinoid cannabidiol (CBD, Fig. 1b). Both have anti-epileptic effects that are thought to result from their activation of the K_V_7.2/7.3 channels [21–23]. We have previously shown that, like retigabine, CBD affects the K_V_7.2–K_V_7.5 channels differently compared to K_V_7.1 and K_V_7.1/KCNE1. Specifically, CBD activates K_V_7.2 and K_V_7.3 by shifting the voltage-dependence of activation to more negative potentials (−ΔV_50_) and activates K_V_7.4 and K_V_7.5 by increasing maximum channel conductance (+Δ*G*_Max_). In a previous study of human K_V_7.1 or K_V_7.1/KCNE1 channels expressed in *Xenopus laevis* oocytes, we found that CBD reduced the *G*_Max_ of K_V_7.1/KCNE1 in a concentration-dependent manner with a concentration causing half-maximal inhibition (IC_50_) of 14 µM, and more potently inhibited the K_V_7.1 channel (IC_50_ = 6 µM) [24]. Given CBD’s distinct pharmacological effects on K_V_7.1 and K_V_7.1/KCNE1 channels compared to K_V_7.2–7.5, a detailed binding site characterisation may offer key molecular insights and guide the development of subtype-selective K_V_7-targeted drugs.

In K_V_7.2, recent cryo-EM structures have resolved that CBD binds at two adjacent binding sites in the PD [13]. One CBD molecule binds in the same site as retigabine, while another one binds just above it (when viewing from the cross-section of the membrane). No structures of CBD bound to K_V_7.1 are currently available. However, we have previously narrowed down the binding site to the PD of K_V_7.1, but were unable to differentiate between several putative binding sites at the time [24]. Moreover, the binding site for CBD in K_V_7.1/KCNE1 remains uncharacterised. To address this, we conducted a thorough binding site investigation using a combination of computational molecular modelling and electrophysiology-based methods. This included using recent innovations in artificial intelligence (AI)–based structural prediction methods to make initial binding site predictions. Then we refined our prediction through molecular docking to a K_V_7.1 structure and a K_V_7.1/KCNE1 homology model and used molecular dynamics (MD) simulations to identify the most stable binding modes among our predictions. To validate our predictions, we utilised site-directed mutagenesis, electrophysiological measurements of human K_V_7.1 and K_V_7.1/KCNE1 channels expressed in *Xenopus laevis* oocytes, and pharmacological co-application experiments. We found that CBD binds in an S5–S6 intrasubunit site in K_V_7.1, and that KCNE1 co-assembly creates a novel binding site for CBD located at the intersubunit interface of S5 and S6 of adjacent K_V_7.1 subunits and the KCNE1 subunit. Intriguingly, this binding site in K_V_7.1/KCNE1 overlaps the canonical retigabine binding site in the K_V_7.2–7.5 channels. These previously uncharacterised CBD binding sites in the K_V_7.1 and K_V_7.1/KCNE1 channels provide insights into the inhibiting effect of CBD on these channels and should aid future efforts to design K_V_7 subtype-selective drugs.

## Materials and methods

### Molecular biology

*Xenopus laevis* frog oocytes were harvested, prepared, and selected in accordance with published procedures [25] and established permits (#1941 and #14515) from the regional ethics board of Linköping, Sweden, or purchased from EcoCyte Bioscience, Germany. Plasmids containing human *KCNE1* and *KCNQ1* were linearized, purified, and in vitro transcribed (T7 mMessage mMachine, Invitrogen) to RNA. For channel mutants, plasmids were mutated via site-directed mutagenesis (QuickChange II XL, Agilent) with mismatched primers (Invitrogen), and then amplified in a commercial *E. coli* bacterial line, XL10-Gold Ultracompetent (Agilent). Nucleic acid concentrations were determined using spectrophotometry (NanoDrop 2000c, Thermo Scientific). Plasmids were sequenced to confirm mutagenesis via Sanger sequencing at the Linköping University Core Facility. RNA was injected into defolliculated oocytes using a microinjector either with 50 ng of K_V_7.1 or a KCNE1-saturating mixture of 25 ng:8 ng K_V_7.1 with KCNE1. For mutants with low current expression, RNA concentrations were doubled. RNA for K_V_7.1 A336G/KCNE1 injection was diluted to a K_V_7.1/KCNE1 ratio of 0.5 ng:8 ng due to high expression after 2 days. Oocytes expressing K_V_7.1 or K_V_7.1/KCNE1 were incubated for 1–3 days at 16 °C, and measured either directly afterwards or kept at 8 °C.

### Electrophysiology

The two-electrode voltage clamp technique was applied to oocytes expressing K_V_7.1 or K_V_7.1/KCNE1 channels after an acclimatising time of 15 min at room temperature in the control extracellular solution. Cells were penetrated with 0.2–1.6 MΩ borosilicate glass tipped Ag/AgCl-electrodes backfilled with 3 M KCl. Signals were amplified with a TEC-10CX (NPI Electronic) amplifier and filtered with a 1 kHz Bessel low-pass filter. Stimulation protocols via Clampex 11.2 software (Molecular Devices) for establishing current traces at differing voltages for K_V_7.1 consisted of a holding voltage (−80 mV), pre-pulse (−100 mV, 2 s), test pulse (−100 mV to +70 mV, +10 mV with each repeat, 3 s) and tail voltage (−30 mV, 1 s), with 17 repeats (15 s sweep-to-sweep interval). For K_V_7.1/KCNE1, the protocol was: holding voltage (−80 mV), test pulse (−80 mV to +80 mV, +10 mV with each repeat, 5 s), and tail voltage (−30 mV, 5 s), with 17 repeats (30 s sweep-to-sweep). For select mutants with shifted voltage dependence, the standard protocol was extended to more positive test pulse voltages (up to +130 mV). The extracellular control solution contained (in mM): 88 NaCl, 1 KCl, 0.4 CaCl_2_, 0.8 MgCl_2_, 15 HEPES, with pH 7.4 set by NaOH. Extracellular control solution was perfused continuously during recordings (1 mL/min). Control solution supplemented with CBD (commercially obtained from Chiron/Sigma-Aldrich or synthesized in-house according to a previous protocol [26] with >98% purity) and/or ML277 (Tocris) was applied using a repeated voltage-step protocol (K_V_7.1: −20 mV for 3 s, K_V_7.1/KCNE1: +40 mV for 5 s) with a holding voltage of −80 mV (sweep-to-sweep: 10 s). CBD and ML277 were dissolved in a 25 mM DMSO stock solution and stored at −20 °C until use. Dilutions of CBD and/or ML277 in control solutions were used immediately after dilution. The application was continued until changes to the current morphology were stabilized or after a maximum of 7 min if no change was observed (time point chosen based on CBD’s application time course). To study CBD effects in each oocyte, a stimulation protocol was applied to determine baseline channel behaviour before the solution exchange, and a stimulation protocol was applied after the solution exchange. Changes to recording conditions are stated where applicable.

### Experimental analysis

Leak compensation was used, or cells were excluded from measurement if the leak was >600 nA. Tail currents (currents at the beginning of the tail pulse, but after the transient spike) were plotted against their preceding test pulse step voltage to establish a tail current *vs* voltage relationship, as an approximation of the conductance *vs* voltage (G(V)) relationship. Data points were fitted with a sigmoidal Boltzmann curve:

$$G\left(V\right)={G}_{{Min}}+\frac{({G}_{{Max}}-{G}_{{Min}})}{[1+{e}^{\frac{{V}_{50}-V}{S}}]}$$

where *G*_Min_ is the minimum conductance, *G*_Max_ is the maximum conductance, *V*_50_ is the voltage generating half maximal conductance, and *s* is the slope of the curve (intrinsic properties of mutants in SI Tables 1, 2). Changes to *G*_Max_ and *V*_50_ were determined for each cell individually from the analysis of baseline tail currents (before CBD exposure) and tail currents after CBD exposure. Δ*G*_Max_ is calculated as % of the current increase/decrease in baseline *G*_Max_, where 0% denotes no change. Δ*V*_50_ is calculated as baseline *V*_50_ subtracted by CBD-exposed *V*_50_, where 0 mV denotes no change. Likewise, Δ*s* denotes a subtraction of the slope measured in the presence of CBD from the slope *s* of the baseline recording.

Concentration-response relationships for CBD or ML277 effects on *G*_Max_ were fitted using a Hill equation:

$$\Delta {G}_{{Max}}=\frac{{E}_{{Max}}}{1+\,{\left(\frac{{C}_{50}}{C}\right)}^{{{{\rm{H}}}}}}$$

where *E*_Max_ is the maximum observed response (i.e. maximal reduction in *G*_Max_ for CBD or maximal increase in *G*_Max_ for ML277, respectively), *C*_50_ is the concentration at which 50% of the response is reached (corresponds to IC_50_ for CBD and EC_50_ for ML277, respectively), *C* is the applied concentration, and H indicates the Hill slope.

For the L266W mutant with and without KCNE1, which showed linearly time-dependent (CBD-independent) current run-up, this was corrected for as previously described [24]. For mutants that showed non-linear time-dependent (CBD-independent) current run-up that may influence the interpretation of CBD effects, putative CBD effects were compared to time-matched control experiments in the absence of CBD and referred to in those cases.

### Statistical analysis

We used Welch’s ANOVA and Dunnett’s T3 to test and compare the effects of CBD on mutant channels relative to wild-type (WT) channels. Student’s *t*-test with Welch’s correction was used for comparison of two experimental conditions. The symbol abbreviations used within the manuscript designating statistical significance are ns: *P* > 0.05, *P < 0.05, **P < 0.01, ***P < 0.001, ****P < 0.0001. *n* denotes the number of oocytes, which was ≥5 for each data set. Every mutant and experimental condition included oocytes from at least two separate batches of oocytes. Experimental data are shown as mean ± SEM if not stated otherwise. The selected representative examples have parameters within one SD of *V*_50_, Δ*G*_Max_, and Δ*V*_50_ of the sample mean.

### Prediction of CBD binding sites in K_V_7 and K_V_7.1/KCNE1

The Chai-1 tool [27] was used to predict models of human K_V_7.1 and K_V_7.1/KCNE1 in complex with four CBD molecules. For the K_V_7.1 subunit, the prediction encompassed residues T104 to Q361, reflecting the first resolved N-terminal residue to the end of the S6 helix in the K_V_7.1 structures [10]. For the KCNE1 subunit, the prediction encompassed residues P35–K69, reflecting the helical portion predicted to interact with the transmembrane domain of K_V_7.1. For K_V_7.1 and K_V_7.1/KCNE1 in complex with CBD, 100 models were generated (20 seeds with 5 model predictions each) and ranked by the interface predicted template (ipTM) score [28] of the CBD molecules. The top-scoring complexes were then selected for simulations.

### Molecular docking

In addition to the CBD-bound models predicted using Chai-1, we generated docking poses for CBD in K_V_7.1 and K_V_7.1/KCNE1. Here, we used the K_V_7.1 structure in the closed pore conformation (PDB: 6UZZ) [10] and constructed a model of K_V_7.1/KCNE1 based on the K_V_7.1/KCNE3 structure in the closed pore conformation (PDB: 6V01) [10]. In brief, 100 models of K_V_7.1/KCNE1 were created and ranked by the intersection of the discrete optimised protein energy (DOPE) and molecular probability density function (MolPDF) scores using the Modeller program [29], and the top-ranking model was selected for docking. The DOPE score is calculated from a statistical potential based on interatomic distances; meanwhile, the MolPDF score is an energy-based metric derived from the sum of all restraints in each model. Both provide a means to assess the quality of a protein model. The sequence lengths of the K_V_7.1 structure and K_V_7.1/KCNE1 model were truncated to match the Chai-1 predictions. A comparison between the K_V_7.1 structure and K_V_7.1/KCNE1 model and their respective Chai-1 predictions is provided (Figure S1). The Kv7.1 structure and K_V_7.1/KCNE1 model were prepared for docking using the protein preparation wizard implemented in Maestro (Schrödinger Release 2022-2: Maestro, Schrödinger, LLC, New York, NY, 2022). This involved adding missing hydrogen atoms and assigning the tautomeric states of titratable residues at pH = 7.0. The CBD molecule was docked using the induced fit docking protocol [30] with the extended sampling option that generates up to 80 docking poses. The docking sites were based on the top-scoring poses from the Chai-1 prediction.

### Molecular dynamics simulations

The top-scoring docked, and Chai-1 poses for CBD in K_V_7.1 and K_V_7.1/KCNE1 were prepared for molecular dynamics simulations. Additionally, we prepared the apo K_V_7.1 structure (PDB: 6UZZ) and K_V_7.1/KCNE1 model based on the K_V_7.1/KCNE3 structure (PDB: 6V00). Each complex and apo protein was placed in a simulation box 140 Å× 140 Å× 100 Å in size using the CHARMM-GUI webserver [31]. This comprised a palmitoyl-2-oleoyl-*sn*-glycero-3-phosphocholine (POPC) lipid bilayer and TIP3P water [32] with 0.15 M KCl solution and excess Cl ions to neutralise the overall charge. The system was described using the CHARMM36M [33] and CHARMM36 (lipids) forcefields; parameters for CBD were generated using CgenFF [34]. Simulations were conducted using the GROMACS program [35], simulation parameters for non-bonded interactions, pressure, and temperature during both equilibration and production followed the CHARMM-GUI defaults [36]. The default CHARMM-GUI equilibration protocol involved gradually withdrawing positional restraints on protein, lipid, and ligand atoms. In the last step, where 50 kJ· mol^−1^ ·nm^−1^ restraints are applied to the protein backbone and ligand heavy atoms, the simulation was extended from 0.5 to 10 ns. Following equilibration, production simulations were conducted for 500 ns in triplicate using a 2 fs timestep.

### Simulation analysis

The root mean square deviation (RMSD) and distance measurement analyses were conducted using the MDAnalysis tool [37]. Clustering analyses were conducted using the “gromos” method [38] implemented in GROMACS, an RMSD cutoff of 2.0 Å was selected for forming clusters. This method works by counting the number of neighbours for each structure using a cut-off distance, selecting the structure with the highest number of neighbours and removing this structure together with its neighbours from the pool of structures. This is repeated until all structures are sorted into clusters; the structure with the highest number of neighbours from each cluster is called the “central structure”. Molecular interactions of CBD within the clusters were calculated using the ProLIF tool [39]. The definitions for counting H-bonds, π-stacking interactions, hydrophobic interactions, and van der Waals (vdW) contacts are provided in Fig. S2. To calculate binding free energies, molecular mechanics/Poisson–Boltzmann surface area (MM/PBSA) calculations were performed using the gmx_MMPBSA tool [40]. Illustrations were made using the PyMOL program (The PyMOL Molecular Graphics System, Version 2.5 Schrödinger LLC). Values reported for analyses of unclustered MD simulations reflect mean ± SD from each of the 4 binding sites of the 3 simulation replicas (*n* = 12).

## Results

### CBD is predicted to bind to the PD of K_V_7.1 and K_V_7.1/KCNE1

To predict potential binding sites for CBD in K_V_7.1 and K_V_7.1/KCNE1, we generated models of CBD in complex with the two proteins using the Chai-1 tool [27]. The top 50 models from a total of 100 were ranked by the ipTM score of the CBD molecules in K_V_7.1 and K_V_7.1/KCNE1 (Fig. 1c, d). When predicted with K_V_7.1 alone, CBD was bound primarily adjacent to the S5–S6 helices of each subunit, which we refer to as the “S5–S6 site”. When predicted with the K_V_7.1/KCNE1 complex, CBD bound primarily at the interface of the KCNE1 subunit and two K_V_7.1 subunits. This site is formed by the S6 helix of the left subunit, the S5’ helix of the right subunit, and the KCNE1 subunit. We refer to this as the “S6–S5’–E1 site”. The top-scoring poses for CBD in complex with K_V_7.1 show that the methyl group of the limonene moiety was positioned between G272 from S5 and A336 from S6, forming hydrophobic interactions with both. The resorcinol ring rested against G272 and formed π-stacking with F275 (Fig. 1e). Furthermore, hydrophobic interactions were observed between the limonene group and F335/F339 and between the hydrophobic tail of CBD and F232/L236. The top-scoring pose for CBD in complex with K_V_7.1/KCNE1 exhibited hydrophobic interactions between the limonene moiety and L266 and F335 from K_V_7.1, and between the resorcinol ring and F53 and F57 from KCNE1. Furthermore, the hydroxy group oxygen of the resorcinol is 4.6 Å from the hydroxy oxygen of S338, suggesting that a small shift in the position of CBD would enable H-bond interactions (Fig. 1f).

The structure of K_V_7.1 has been resolved in complex with the activator ML277, which binds at a PD-spanning site [17]. The availability of ML277-bound K_V_7.1 structures enables comparison to the proposed CBD binding mode in K_V_7.1. This revealed that the predicted CBD binding mode partially overlapped with ML277 in K_V_7.1 (Fig. 1g). To test whether CBD and ML277 indeed use the same binding pocket, we conducted competition experiments between CBD and ML277 (Fig. 1h). For ML277 alone, clear effects were seen at 1 µM as an increase of *G*_Max_ by 193% ± 28%. In contrast, for cells pretreated and co-exposed with 30 µM CBD, *G*_Max_ was only increased by 18% ± 3% by 1 µM ML277 (Fig. 1h). In the presence of 30 µM CBD, the EC_50_ of ML277 was shifted by about two orders of magnitude, suggesting that CBD and ML277 compete for the same binding site, in accordance with our predictions.

### K_V_7.1 mutagenesis corroborates CBD-interacting residues in the S5–S6 site

**Fig. 2:**
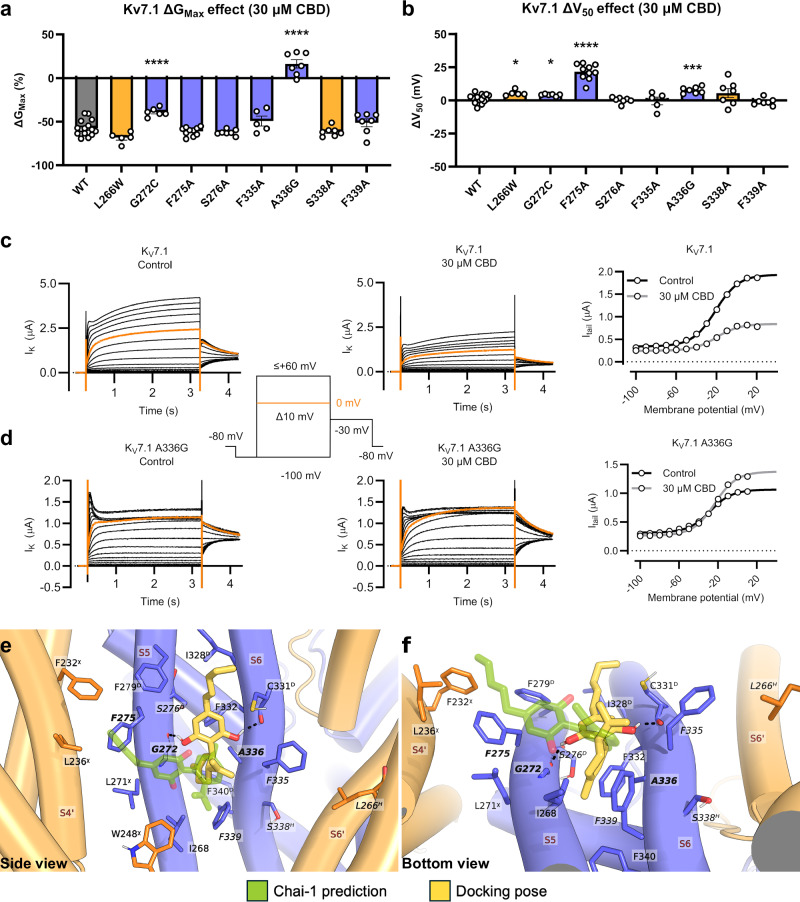
CBD binds to an intrasubunit pocket in the PD of K_V_7.1. Change in maximum conductance (Δ*G*_Max_, **a**) or shift in voltage-dependence of channel activation (Δ*V*_50_, **b**) induced by 30 µM CBD for indicated human K_V_7.1 mutants. Experiments were performed in *Xenopus laevis* oocytes expressing K_V_7.1 alone. Data displayed as mean ± SEM. *n* = 5–16 oocytes, with individual data points shown. Statistics denote Dunnett’s T3 multiple comparison test in relation to WT effect, with *P < 0.05, ***P < 0.001, ****P < 0.0001. ns > 0.05 not shown. **c**, **d** Representative current traces of WT or indicated mutant under control conditions or in the presence of 30 µM CBD, and their respective G(V) curve. Orange sweep represents the current generated by a test pulse to 0 mV. The inset shows the voltage protocol. For these cells: WT: *V*_50, Ctrl_ = −21 mV, *V*_50, CBD_ = −21 mV, *s*_Ctrl_ = 12.0 mV, *s*_CBD_ = 8.8 mV, *G*_Max, Ctrl_ = 1.95 µA, *G*_Max, CBD_ = 0.81 µA; K_V_7.1 A336G: *V*_50, Ctrl_ = −28 mV, *V*_50, CBD_ = −23 mV, *s*_Ctrl_ = 11.7 mV, *s*_CBD_ = 10.8 mV, *G*_Max, Ctrl_ = 1.06 µA, *G*_Max, CBD_ = 1.37 µA. Docked poses of CBD in K_V_7.1 as viewed from the cross-section of the membrane (side view, **e**) and as viewed from the cytoplasm (bottom view, **f**). See Fig. 1 for details on subunit colouring. Residues adjacent to either CBD pose are shown, a “χ” and “D” superscript is used to denote residues within 4 Å of Chai-1 predicted CBD or docked CBD poses, respectively. Residues beyond 4 Å of either have a “H” superscript, and residues within 4 Å of both do not have a superscript. Residues that were tested using site-directed mutagenesis are italicised, and residues with a significant difference compared to WT are bolded as well. The overlayed docked and Chai-1 predicted poses of CBD are shown in yellow and faded green, respectively; the black dashed lines represent H-bonds.

To test the validity of the proposed binding site in K_V_7.1, we mutated residues predicted to interact with CBD in the S5–S6 site. Given that CBD was predicted to interact with two opposing residues between the S5 and S6 helices, G272 and A336, respectively, we mutated these residues. We hypothesised that changing G272 to a bulkier residue, cysteine (G272C), may reduce the space available for CBD to bind. G272C had previously been reported to abolish ML277 effects [18, 41]. In line with this, the G272C mutation decreased the *G*_Max_ reduction induced by 30 µM CBD, from −57% ± 2% for WT to −39% ± 2% for G272C (Fig. 2a, Figure S3 for representative examples of CBD effects on mutants), due to a decrease in CBD’s efficacy for G272C (Fig. S4). For A336, we hypothesized that a smaller residue, glycine (A336G), may increase the space for CBD to bind. Strikingly, we observed a complete removal of the inhibitory effect of CBD on *G*_Max_ for 30 µM CBD (Δ*G*_Max_ = +16% ± 5%, Fig. 2a, Fig. 2c, d for a representative example of CBD effect on WT and the A336G mutant), and a smaller inhibition than WT for 100 µM CBD (highest concentration tested, WT: Δ*G*_Max_ = −62% ± 14% vs. A336G: Δ*G*_Max_ = −18% ± 6%). The small apparent increase in *G*_Max_ for 30 µM was not significantly different from time-matched control experiments (Fig. S5).

We also generated mutants of other residues in the S5–S6 site. This included mutating F275, predicted to form π-stacking interactions with CBD, to alanine (F275A). This mutation did not change the *G*_Max_ effect of 30 µM CBD (Δ*G*_Max_ = −58 ± 3%), but induced a clear shift in the voltage dependence of channel activation (*V*_50_), which was not seen in WT (from Δ*V*_50_ = +1.4 ± 0.8 mV for WT to +21.6 ± 1.9 mV for F275A, Fig. 2b). Adjacent to F275, we tested whether the hydroxy sidechain of S276 could form an H-bond with CBD by mutating it to S276A. The mutation was not found to impact the CBD response compared to WT (Fig. 2a, b). Adjacent to A336 is F335, which formed hydrophobic interactions to CBD. Mutating F335 did not change the CBD response compared to the WT when mutated to alanine (Fig. 2a, b). The WT-like response of F335A was in contrast to our previous study [24], which is likely a result of varied CBD loss in different experimental systems (see Discussion for further details). Additionally, we tested F339, located one helical turn below F335 and adjacent to the limonene group of CBD. Mutating this residue to alanine did not appear to alter CBD response compared to WT.

To verify the absence of CBD binding to the S6–S5’–E1 site in K_V_7.1 alone, we assessed the CBD effect on S338A (S6) and L266W (S5). Neither mutant changed the *G*_Max_ effect of 30 µM CBD (Fig. 2a). In summary, the site-directed mutagenesis experiments suggest that several residues in the S5–S6 site (G272, F275, A336) are important for CBD binding or effects on K_V_7.1 alone, while residues in the S6–S5’–E1 site are not.

After validating that the S5–S6 site is important for binding CBD in K_V_7.1, we generated alternative docking poses for CBD at this site using an induced fit docking protocol. This protocol samples alternative residue conformations in the binding site and generates docking poses not otherwise accessible with the original residue conformations. Here, we used the K_V_7.1 structure in the closed pore conformation (PDB: 6UZZ) [10]. The top-scoring docking pose shows that CBD is able to bind deeper into the binding pocket than predicted by the Chai-1 tool (Fig. 2e, f). Like what we concluded after using Chai-1 prediction, the predicted CBD docking pose is only compatible with the absence of ML277 in the binding site (Fig. S6). The docking pose of CBD is oriented vertically, lining the groove between the S5 and S6 helices, while in the Chai-1 prediction, CBD is perpendicular to the helices. The vertical CBD binding pose predicted through flexible docking is supported by H-bond interactions from the hydroxy groups of the resorcinol to the backbone carbonyl oxygens of G272 and C331. The limonene group of CBD penetrates deeper into the pocket between G272 and A336 (Fig. 2e, f). Both in the Chai-1 and docking poses, the limonene group of CBD forms hydrophobic interactions with F335/F339. This alternative binding pose shifts the aliphatic tail away from F232, L236, and F275 compared to the Chai-1 prediction. We explore the implications of this through MD simulations in a subsequent section.

### K_V_7.1/KCNE1 mutagenesis corroborates CBD interacting primarily with residues in the S6–S5’–E1 site

**Fig. 3:**
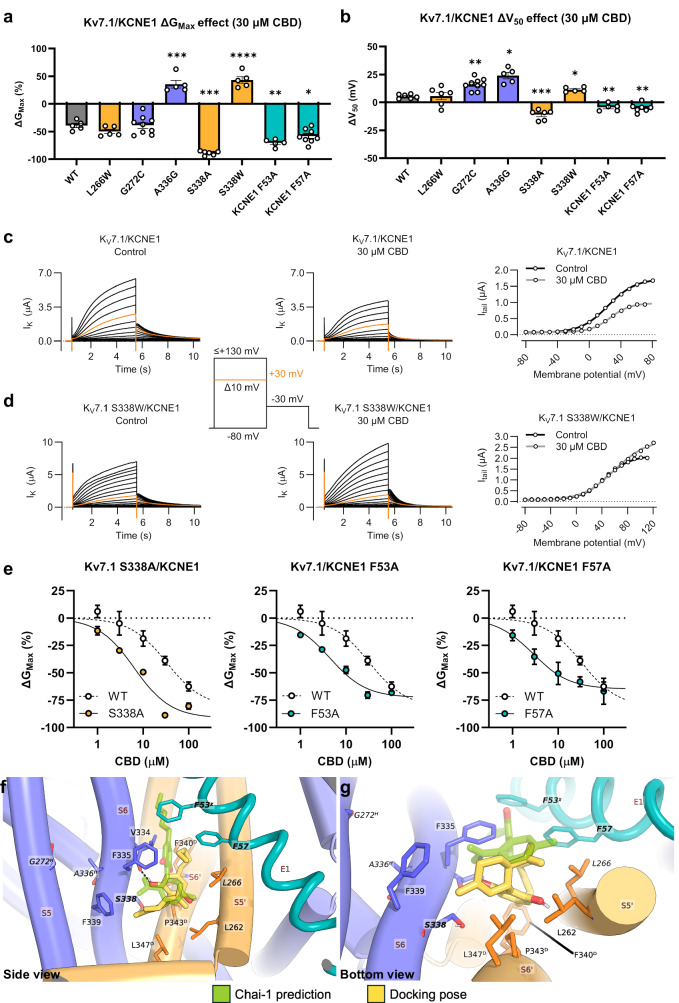
CBD binds to a distinct intersubunit PD pocket formed by K_V_7.1 and KCNE1. Change in maximum conductance (Δ*G*_Max_, **a**) or shift in voltage-dependence of channel activation (Δ*V*_50_, **b**) induced by 30 µM CBD for indicated human K_V_7.1 mutants and/or KCNE1 mutants. Experiments were performed in *Xenopus laevis* oocytes expressing K_V_7.1/KCNE1. Data displayed as mean ± SEM. *n* = 5–7 oocytes, with individual data points shown. Statistics denote Dunnett’s T3 multiple comparison test in relation to WT effect, with *P < 0.05, **P < 0.01, ***P < 0.001, ****P < 0.0001. ns > 0.05 not shown. **c**, **d** Representative current traces of WT or indicated mutant under control conditions or in the presence of 30 µM CBD, and their respective G(V) curve. Orange sweep represents the current generated by a test pulse to +30 mV. Inset shows voltage protocol. For these cells: WT: *V*_50, Ctrl_ = +24 mV, *V*_50, CBD_ = +27 mV, *s*_Ctrl_ = 17.1 mV, *s*_CBD_ = 12.4 mV, *G*_Max, Ctrl_ = 1.73 µA, *G*_Max, CBD_ = 0.98 µA; K_V_7.1 S338W/KCNE1: *V*_50, Ctrl_ = +46.0 mV, *V*_50, CBD_ = +59.2 mV, *s*_Ctrl_ = 19.3 mV, *s*_CBD_ = 24.6 mV, *G*_Max, Ctrl_ = 2.12 µA, *G*_Max, CBD_ = 2.83 µA. **e** Concentration-response relationship for CBD effect on *G*_Max_ of indicated mutants (filled symbols). Data for WT K_V_7.1/KCNE1 is included for comparison (dotted line and open symbols). Data displayed as mean ± SEM. *n* = 5–6 oocytes. Best fits: K_V_7.1/KCNE1 WT: *E*_Max_ (i.e. maximal reduction in *G*_Max_ induced by CBD) = –81%; IC_50_ = 32.6 µM (CI: 10.4–617, *R*^2^ = 0.7858); K_V_7.1 S338A/KCNE1: *E*_Max_ = –92%; IC_50_ = 6.4 µM (CI: 3.45–13.6, *R*^2^ = 0.9048), K_V_7.1/KCNE1 F53A: *E*_Max_ –73%; IC_50_ = 4.4 µM (CI: 2.0–10.4,* R*^2^ = 0.9091), and K_V_7.1/KCNE1 F57A: *E*_Max_ = –65%; IC_50_ = 2.8 µM (CI: 1.1–7.48, *R*^2^ = 0.5276). Note that the IC_50_ for WT was higher than reported in Pökl et al. [24], likely due to slight differences in experimental perfusion systems (see Discussion). Docked poses of CBD in K_V_7.1/KCNE1 as viewed from the cross-section of the membrane (side view, **f**) and as viewed from the cytoplasm (bottom view, **g**). See Fig. 1 for details on subunit colouring. Residues adjacent to either CBD pose are shown, a “χ” and “D” superscript is used to denote residues within 4 Å of Chai-1 predicted CBD or docked CBD poses, respectively. Residues beyond 4 Å of either have a “H” superscript, and residues within 4 Å of both do not have a superscript. Residues that were tested using site-directed mutagenesis are italicised, and residues with a significant difference compared to WT are bolded as well. The overlayed docked and Chai-1 predicted poses of CBD are shown in yellow and faded green, respectively; the black dashed lines represent H-bonds.

As with K_V_7.1, we experimentally tested the binding site prediction for CBD in K_V_7.1/KCNE1. Firstly, we examined S338, which is near CBD in the K_V_7.1/KCNE1 model and was observed to have no impact on the CBD effect in K_V_7.1 alone. Unlike in K_V_7.1, in K_V_7.1/KCNE1 the S338A mutant yielded a channel that was more strongly inhibited by 30 µM CBD than WT (Δ*G*_Max_ = −89% ± 12% for S338A compared to −39% ± 4% for WT, Fig. 3a, Figure S7 for representative examples of CBD effects on mutants) and responded to CBD with a more pronounced *V*_50_ shift (Δ*V*_50_ = −11.1 ± 1.8 mV for S338A compared to +5.4 ± 0.8 mV for WT, Fig. 3b). We hypothesised that a much bulkier residue at position 338, tryptophan (S338W), would displace CBD from the site. In line with this, the S338W mutant was not inhibited by 30 µM CBD (Δ*G*_Max_ = +43% ± 6%, Fig. 3a, Fig. 3c, d for a representative example of CBD effect on WT and the S338W mutant) or 100 µM CBD (Δ*G*_Max_ = +20% ± 11%). The apparent increase in *G*_Max_ was not significantly different from time-matched control experiments (Fig. S5).

To assess if KCNE1 contributes to the S6–S5’–E1 CBD binding site, we tested the KCNE1 mutants F53A and F57A, both predicted to form hydrophobic interactions to CBD. The F53A mutant was more strongly inhibited by 30 µM CBD than WT (Δ*G*_Max_ = −70% ± 3%, Fig. 3a). Likewise, the F57A mutant was more strongly inhibited by 30 µM CBD than WT (Δ*G*_Max_ = −58% ± 5%, Fig. 3a). Like for the S338A mutant, 30 µM CBD shifted *V*_50_ to more negative voltages for the F53A and F57A mutants (Δ*V*_50_ = −4.4 ± 1.5 mV, and −5.4 ± 0.8 mV, respectively, Fig. 3b). Despite the proximity of CBD to L266 in the S6–S5’–E1 site, the bulkier L266W mutant, which was intended to prevent CBD binding, did not alter the CBD response in K_V_7.1/KCNE1 (Fig. 3a, b). For Kv7.1/KCNE1 mutants with enhanced response to 30 µM CBD, we assessed CBD effects across a broader range of concentrations to get further insights into how the mutants alter the pharmacology. All alanine mutants in the S6–S5’–E1 CBD binding site clearly increased CBD’s potency by about one order of magnitude (Fig. 3e).

To verify that CBD did not bind at the S5–S6 site in K_V_7.1/KCNE1, we assessed the CBD effect on G272C co-expressed with KCNE1. The G272C mutant did not change the *G*_Max_ effect of 30 µM CBD in K_V_7.1/KCNE1 (Fig. 3a); however, a *V*_50_ response to CBD was observed (Δ*V*_50_ = +16.1 ± 1.7 mV, Fig. 3b). Hence, in K_V_7.1/KCNE1, the G272C mutant preserved the *G*_Max_ response to CBD and displayed a small, additional *V*_50_ effect. This suggests that CBD may exhibit some binding to the S5–S6 site in addition to the S6–S5’–E1 site. Interestingly, the A336G mutant, when co-expressed with KCNE1, resulted in a complete abolishment of the *G*_Max_ reduction normally induced by 30 µM CBD (Δ*G*_Max_ = +36% ± 7%, Fig. 3a, which was a significant increase in *G*_Max_ compared to time-matched experiments, Fig. S5) and induced a more pronounced *V*_50_ shift (Δ*V*_50_ = +24.0 ± 2.9 mV, Fig. 3b). Moreover, there was no *G*_Max_ reduction induced by 100 µM CBD for the A336G mutant (Δ*G*_Max_ = +5.6% ± 8.0%). We note that A336 is only two residues away from S338 in the S6 helix, indicating that A336 may contribute to both the S5–S6 site and the S6–S5’–E1 site. In summary, the site-directed mutagenesis experiments suggest that some residues in the S6–S5’–E1 site (S338 on K_V_7.1 and F53 and F57 in KCNE1) are important for CBD binding or effects in K_V_7.1/KCNE1. However, unlike K_V_7.1, where mutations to residues in the S6–S5’–E1 site did not affect the CBD response, in K_V_7.1/KCNE1, mutation of G272 at the S5–S6 site did impact the CBD response.

As with K_V_7.1, we generated alternative docking poses for CBD binding in K_V_7.1/KCNE1 using an induced fit docking protocol (Fig. 3f, g). We constructed a model of K_V_7.1/KCNE1 based on the K_V_7.1/KCNE3 structure in the closed pore conformation (PDB: 6V00) [10]. The docking pose of CBD is deeper into the S6–S5’–E1 pocket compared to the Chai-1 prediction, similar to what we observed in K_V_7.1. As such, additional hydrophobic interactions are established to S6’ helix residues, between the hydrophobic tail and F340, between the resorcinol ring and P343, and between the limonene group and L347. Relative to the Chai-1 prediction, the resorcinol ring is rotated by ~90°, meanwhile the limonene group is flipped by 180°. This prediction enables CBD to pack closer to S338 and enables F335 and KCNE1 residue F53 to come closer to each other and form a “seal” around the aliphatic tail of CBD.

### MD simulations illustrate the stability of CBD poses

**Fig. 4:**
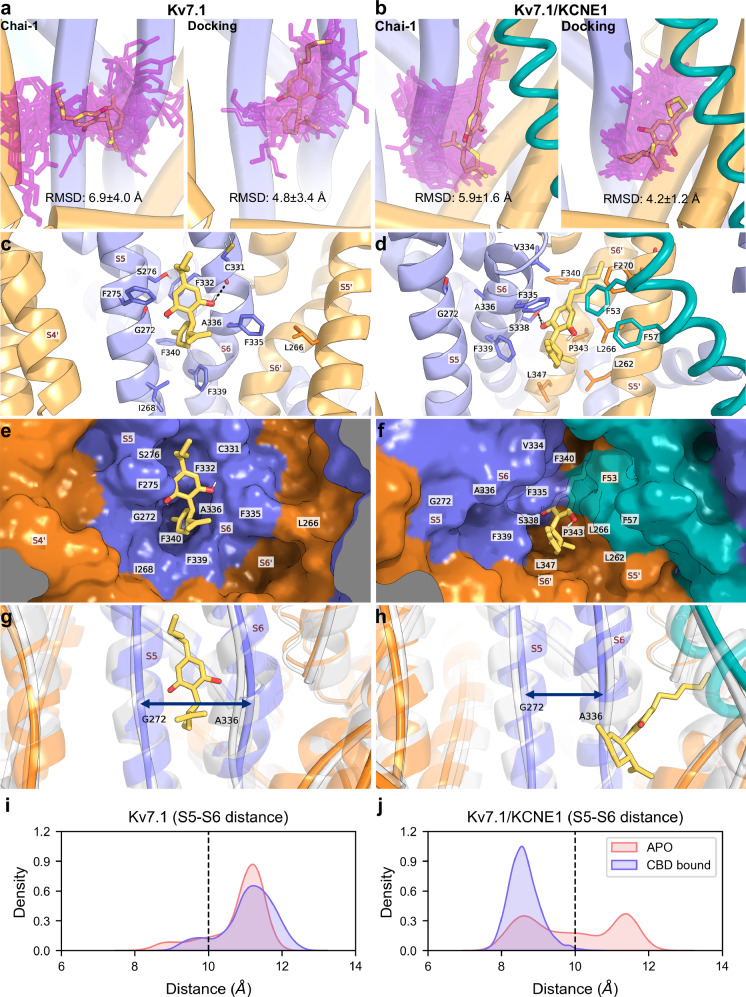
Molecular dynamics simulations of CBD in human K_V_7.1 and K_V_7.1/KCNE1 show binding site properties. Ensemble of CBD poses from simulations of the Chai-1 predicted and docking poses, in K_V_7.1 (**a**) and K_V_7.1/KCNE1 (**b**). The ensemble of poses is shown in faded purple representation and overlayed over the initial pose in yellow, 10 simulation frames are sampled from 300–500 ns during each simulation replica (30 total in each panel). RMSD ± SD values were calculated for CBD relative to the initial pose from each of the 4 binding sites of the 3 simulation replicas (*n* = 12). Representative binding poses obtained from largest binding pose clusters of CBD in K_V_7.1 (**c**) and K_V_7.1/KCNE1 (**d**), residues adjacent to CBD are displayed, the black dashed lines represent H-bonds. Surface representation of protein from binding poses above in K_V_7.1 (**e**) and K_V_7.1/KCNE1 (**f**), the surfaces of F335 (S6) and F53 (E1) are faded to show CBD behind them. Illustration of the S5–S6 interhelical distance in the CBD-bound K_V_7.1 (**g**) and K_V_7.1/KCNE1 (**h**). The representative simulation frames with the coloured helices reflect the binding pose clusters of CBD, which are overlayed on the initial protein conformation, where the helices are coloured white. The arrow in dark blue colour indicates where the S5–S6 distance was measured. See Fig. 1 for details on subunit colouring. Distance distribution between the S5–S6 helices in K_V_7.1 (**i**) and K_V_7.1/KCNE1 (**j**).

To test the stability of the CBD poses predicted from Chai-1 and docking, we conducted triplicate MD simulations lasting for 500 ns in each binding site. First, the stability of the protein was assessed using the RMSD of Cα atoms relative to the initial protein conformation. The plots demonstrate that the protein Cα atom positions were stable by 300 ns with an overall deviation of around 3.0 Å from the initial structure, except in the Chai-1 predicted K_V_7.1/KCNE1 protein structure, which was shifted by around 4.0 Å (Figure S8). We therefore present binding pose and interactions for CBD from 300–500 ns to avoid frames where the protein was still equilibrating. The stability of CBD was quantified by average RMSD and visually, by overlaying simulation frames on the initial protein conformations (Fig. 4a, b). Both RMSD and the simulation frames show that CBD had more stable binding modes for simulations initiated from docking poses compared to the Chai-1 predicted poses. In K_V_7.1, the simulation frames show that CBD simulations initiated from the Chai-1 prediction sampled a variety of poses that spanned the PD (RMSD = 6.9 ± 4.0 Å); meanwhile, the simulation initiated from the docking pose sampled more uniform poses (RMSD = 4.8 ± 3.4 Å), mostly around the S5 and S6 helices. In K_V_7.1/KCNE1, the simulation initiated from the Chai-1 prediction shows that CBD drifted further from the binding pocket under the KCNE1 subunit (RMSD = 5.9 ± 1.6 Å) compared to the simulation initiated from the docking pose (RMSD = 4.2 ± 1.2 Å).

We tracked the distance between CBD and residues known to influence its effect in K_V_7.1 (G272, F275, A336) and K_V_7.1/KCNE1 (S338, and KCNE1 residues F53 and F57) (Fig. S9). In K_V_7.1, for the majority of the simulation, CBD remained overall closer to G272, F275, and A336 in the simulation initiated from the docking pose compared to the Chai-1 pose. The reason is that throughout the simulation initiated from the Chai-1 prediction, CBD drifts away from the three binding site residues (distance >= 10 Å). We note, however, that for the simulation initiated from the docking pose, CBD leaves the binding site in one simulation replica around 400 ns. In contrast to the shallower S5–S6 site of K_V_7.1, in the deeper S6–S5’–E1 K_V_7.1/KCNE1 site, CBD did not leave the binding site in any simulation replicas. We observed that in the Chai-1–initiated pose, CBD moved away from S338 but remained close to F53 across most simulation replicas, while the docked pose showed the opposite trend—drifting from F53 but staying near S338. Both poses stayed similarly close to F57. Because S338 lies at the base of the S6–S5’–E1 site pocket, the Chai-1–initiated pose shifted away from the base of the pocket, but in doing so came closer to F53 than the docked pose.

To investigate how the docked and Chai-1 CBD poses affected binding free energies, we performed MM/PBSA calculations. Interestingly, we found that CBD had similar binding free energy in K_V_7.1, regardless of whether the simulation was initiated from the docked or Chai-1 predicted pose (−24.3 ± 4.0 and −21.2 ± 4.5 kcal/mol, respectively). A greater difference was observed in K_V_7.1/KCNE1 where the simulations initiated from the docked pose had a more favourable energy of −27.5 ± 3.2 kcal/mol compared to the one from Chai-1 at −22.9 ± 2.0 kcal/mol.

In summary, we have compared the differences in CBD binding in terms of RMSD, distance to binding site residues, and binding free energy in K_V_7.1 and K_V_7.1/KCNE1. We found that in K_V_7.1, the shallower S5–S6 site enabled CBD to drift away from the site. Despite the pose initiated from docking being overall more stable in terms of RMSD and distance to binding site residues, this did not translate into a meaningful difference in binding free energy between the Chai-1 and docked poses. In contrast, in the deeper S6–S5’–E1 site of K_V_7.1/KCNE1, the pose initiated from docking, which remained closer to the base of the site (S338), had a more favourable binding free energy compared to the pose initiated from the Chai-1 prediction, which drifted away from the base of the site.

### MD simulations illustrate CBD molecular interactions and binding pocket dynamics

We performed clustering analysis to find the most representative binding pose of CBD in K_V_7.1 and K_V_7.1/KCNE1 from simulations initiated from the docked conformations (Figs. 4c and 4d, respectively). We note that the simulation frames illustrated in Fig. 4 are based on the “central structure” (see Methods), which is the most representative of each cluster [38], but a spectrum of simulation frames is provided in Fig. S10 to highlight the distribution of conformations. The most populated simulation cluster in K_V_7.1 resembled the initial docked pose and enabled the resorcinol ring of CBD to remain within the gap between S5 (G272) and S6 (A336). However, the resorcinol ring could only maintain the H-bond interaction with the backbone carbonyl of C331 and not G272 (Fig. 4c). We quantified the percentage of cluster frames where this H-bond interaction was present, showing that CBD interacted with G272 and C331 in ~2% and ~93% of cluster frames, respectively. Analysis of other prominent molecular interactions (present in > 33% of cluster frames) revealed that the resorcinol group of CBD formed vdW/hydrophobic interactions with G272 and F275 as well as π-stacking interactions with F332. Further, the limonene group formed hydrophobic interactions with A336, in addition to I268, F335, and F339, see Fig. S2 for details. The proposed binding mode of CBD in K_V_7.1 is supported by agreement between the simulation and site-directed mutagenesis findings, suggesting a role for G272, F275, and A336 in CBD binding. However, given prominent interactions with F335 and F339, it is unclear why mutating these residues to alanine did not affect response to CBD.

Similarly, in K_V_7.1/KCNE1, the most populated simulation cluster for CBD resembled the initial docking pose. However, the resorcinol ring had shifted to break the H-bond with the backbone carbonyl of V334 and instead formed interactions with the sidechain hydroxy group of S338 (Fig. 4d). This interaction was present in ~95% of the cluster frames. Analysis of other prominent interactions (present in > 33% of cluster frames) revealed that in addition to KCNE1 residues F53 and F57, F335 formed hydrophobic interactions with the resorcinol ring of CBD. The limonene group formed hydrophobic interactions with L262, F339, P343, and L347. Furthermore, the CBD aliphatic tail formed other hydrophobic interactions, including with L266 (Fig. S2). The proposed binding mode for CBD in K_V_7.1/KCNE1 is supported by agreement between the simulation and site-directed mutagenesis findings, suggesting a role for S338 and KCNE1 residues F53 and F57 in CBD binding. However, given the proximity to L266, it is unclear why mutating it to the bulkier tryptophan did not affect response to CBD.

A surface representation of the most populated clusters is provided to further characterise the S5–S6 site of K_V_7.1 and the S6–S5’–E1 site of K_V_7.1/KCNE1. In K_V_7.1, we observe that G272 and A336 form a shallow pocket that houses the limonene group of CBD (Fig. 4e). F275 partially forms a cover over the resorcinol ring of CBD; this conformation was present in 92% of cluster frames (see Fig. S10 for the spectrum of simulation frames). Meanwhile, in K_V_7.1/KCNE1, CBD is inside a pocket behind the KCNE1 subunit; the residue F335 and the KCNE1 residue F53 can interact, forming a “cover” over the resorcinol ring of CBD (Fig. 4f). We note, however, that F53 exhibited a variety of conformations such that CBD was “covered” in around 66% of cluster frames (Figure S10b). In addition to contributing potentially favourable molecular interactions to CBD, the KCNE1 subunit may stabilise the conformations of binding site residues in the S6–S5’–E1 site of K_V_7.1/KCNE1. Specifically, KCNE1 subunit residues F53 and F57 seemed to stabilise the conformations of residues F270 and L266, in K_V_7.1/KCNE1 (see Fig. S10c for details).

We investigated whether the binding of CBD to the S6–S5’–E1 site influences the S5–S6 site. To achieve this, we measured the distance between the helical cores of the S5 and S6 helices centred around G272 and A336, respectively (Fig. 4g–h). In K_V_7.1, the distance distribution was similar regardless of the presence of CBD (Fig. 4i). The pocket remained open with an average interhelical distance of 10.8 ± 0.7 Å without CBD and 11.1 ± 0.7 Å with CBD. By contrast, in K_V_7.1/KCNE1, without CBD, the S5–S6 helical distance varied (Fig. 4j), sampling closed and open conformations of the pocket (interhelical distance of 9.9 ± 1.3 Å on average). With CBD bound to the S6–S5’–E1 site, the helices are mainly packed together and close the pocket (interhelical distance of 8.6 ± 0.3 Å on average). The opening distance from each replica/binding site is compared to the initial opening distance in Fig. S11. We observe that only with CBD in K_V_7.1/KCNE1, all replicas shifted towards a smaller, more closed interhelical distance.

In summary, we obtained the most prominent binding poses of CBD in K_V_7.1 and K_V_7.1/KCNE1 through clustering analyses of the simulations. Consistent with site-directed mutagenesis, CBD interacted with residues critical for its activity: G272, F275, and A336 in K_V_7.1, and S338, along with KCNE1 residues F53 and F57 in K_V_7.1/KCNE1. CBD also interacted with residues whose mutation did not alter activity, including F335 and F339 in K_V_7.1 and L266 in K_V_7.1/KCNE1. Surface representations showed that the S6–S5’﻿–E1 binding site of K_V_7.1/KCNE1 forms a deep pocket sealed by F335 and KCNE1 residue F53, whereas the S5–S6 site in K_V_7.1 is comparatively shallow and open. The KCNE1 subunit not only contributes to the S6–S5’﻿–E1 pocket but also influences S5–S6 site dynamics, reducing its accessibility, particularly when CBD is bound to K_V_7.1/KCNE1.

### Sequence and structure comparison of CBD pockets elucidates K_V_7 selectivity

**Fig. 5:**
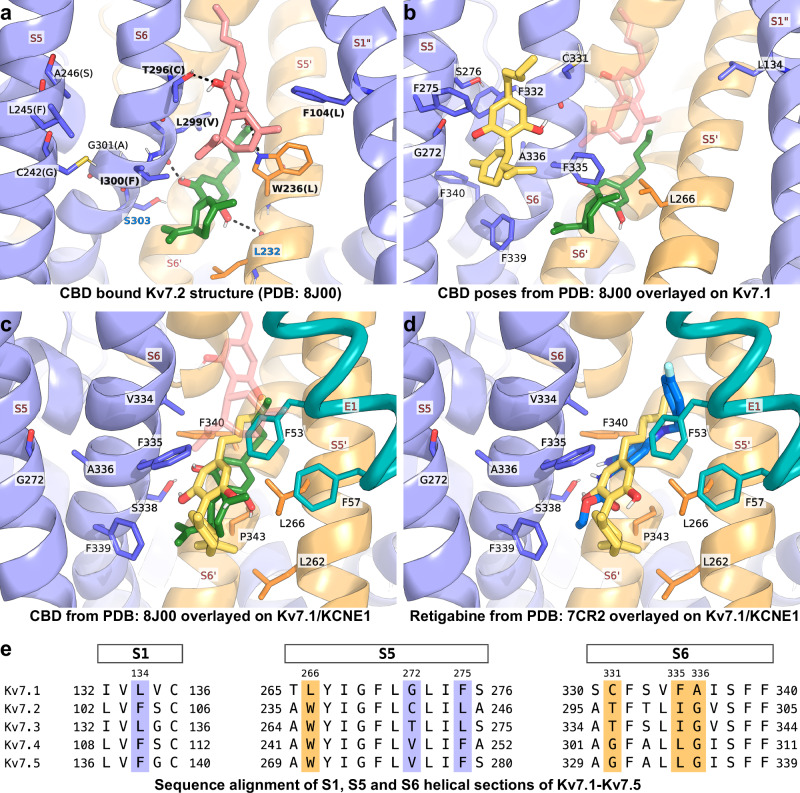
CBD binding comparison between human K_V_7.1 and K_V_7.2, and sequence comparison show K_V_7 subtype differences. **a** The CBD-bound K_V_7.2 structure in the closed pore conformation (PDB 8J00) [13], with light red and dark green colouring for the upper and lower CBD molecules, respectively. Only residues near the CBD molecules or the S5–S6 site and that are different between K_V_7.1 and K_V_7.2 are displayed (with the exception of K_V_7.2 residue S303). Labels reflect the K_V_7.2 residue and numbering, and the K_V_7.1 residue appended to the end (i.e., W236(L) reflects that K_V_7.1 has a leucine in place of the tryptophan), L232 and S303 are coloured in blue to highlight that they are each conserved between K_V_7.1 and K_V_7.2 (and all other K_V_7s). Residues that interact with CBD are bolded. **b** Representative CBD binding pose (yellow) from the largest binding pose cluster in K_V_7.1 (S5–S6 binding site), which is overlayed on the CBD-bound K_V_7.2 structure. Representative CBD binding poses (yellow) from the largest binding pose cluster in K_V_7.1/ KCNE1 (S6–S5’–E1 binding site), which is overlayed on the CBD-bound K_V_7.2 structure (**c**) and retigabine-bound K_V_7.2 structure (PDB: 7CR2) [12] (retigabine is coloured in blue) (**d**). In **b**–**d**, the K_V_7.2 structures are hidden except for CBD and retigabine. The upper CBD molecule in (**b**) and (**c**) is faded for clarity. See Fig. 1 for details on subunit colouring. **e** Sequence alignment showing sections of the S1, S5, and S6 helices. The coloured blocks indicate key CBD interacting residues with K_V_7.1 (our prediction) or K_V_7.2 (structure) and their conservation across K_V_7 subtypes, purple-blue and orange colour blocks are matched to the protein colours in the panels above.

The recently published K_V_7.2 structure in complex with CBD [13] enables a comparison of the binding modes proposed in different isoforms. The CBD-bound K_V_7.2 structure in the closed pore state (PDB: 8J00) [13] displays two bound CBD molecules (Fig. 5a). Except for K_V_7.2 residues L232 and S303, only residues that are different between K_V_7.2 and K_V_7.1 are displayed. The lower CBD molecule (dark green) is positioned below the K_V_7.2 residue W236, which corresponds to L266 in K_V_7.1. The K_V_7.2 residue I300 forms hydrophobic interactions with the limonene group of both CBD molecules and corresponds to the bulkier K_V_7.1 residue F335. Superimposing the CBD molecules bound in the K_V_7.2 structure on our CBD-bound K_V_7.1 prediction reveals that in K_V_7.1, L266 and F335 sterically hinder the lower binding mode of CBD in K_V_7.2 (Fig. 5b). On the other hand, the upper CBD molecule (light green) forms H-bonds through the resorcinol hydroxyls with W236 and T296 in K_V_7.2 (K_V_7.2 residue T296 corresponds to C331 in K_V_7.1). Further, CBD, through its limonene group, forms hydrophobic interactions with K_V_7.2 residue F104 (which corresponds to L134 in K_V_7.1). Therefore, these residue differences, which remove important molecular interactions, rationalise why CBD likely does not exhibit the upper binding pose in K_V_7.1 that is seen in the K_V_7.2 structure.

On the other hand, there are several differences around the S5–S6 pocket in K_V_7.2 compared to K_V_7.1. In this pocket, all three K_V_7.1 residues (G272, F275 and A336) shown to be important for the effect of CBD are different in K_V_7.2 (C242, L245, and G301, respectively). We have shown that the mutants G272C and A336G, mirroring the K_V_7.2 substitutions, reduce the CBD response of K_V_7.1; therefore, CBD is unlikely to bind in a similar pose in K_V_7.2.

Alignment of the CBD-bound K_V_7.2 structure to the binding site prediction for K_V_7.1/KCNE1 shows that the CBD pose in the S6–S5’–E1 site resembles the lower CBD binding pose in K_V_7.2 (Fig. 5c). This contrasts with the K_V_7.1 structure, where L266 and F335 sterically hinder CBD from binding. In addition to CBD in K_V_7.2, CBD at the S6–S5’–E1 site also overlaps with retigabine from K_V_7 structures, displayed as an overlay from the K_V_7.2 structure (PDB: 7CR2 [12], Fig. 5d). This suggests that the addition of KCNE1, specifically KCNE1 residue F53, creates a retigabine-like site in K_V_7.1/KCNE1.

Expanding the sequence comparison of regions involved in CBD binding to all other K_V_7 subtypes (Fig. 5e) shows that K_V_7.1 residue L134 is homologous to K_V_7.3 residue L134, and K_V_7.1 residue F275 is homologous to K_V_7.4 residue F251 and K_V_7.5 residue F279. For the other highlighted residues, K_V_7.1 is unique among the K_V_7 family. In this sense, the S5–S6 site may be unique to K_V_7.1 as it is the only channel in the family with a glycine (S5) and alanine (S6) in the positions G272 and A336. Furthermore, K_V_7.1’s unique residues L266 and F335 seem to occlude access to the retigabine/CBD binding site of other K_V_7 channels. However, our findings suggest that inclusion of the KCNE1 subunit creates a binding mode where CBD partly overlaps the retigabine/CBD binding modes found in other K_V_7 channels.

## Discussion

The molecule CBD uniquely inhibits the K_V_7.1 and K_V_7.1/KCNE1 channels while having activating effects on neuronal K_V_7.2–K_V_7.5 channels [24]. As such, investigating the CBD binding mode that underlies these effects is important from the perspective of developing subtype-selective drug compounds. In this study, using a combination of computational and experimental approaches, we propose that CBD binds to sites that are not only different between the K_V_7.1 and K_V_7.1/KCNE1 channels but also different compared to the neuronal K_V_7 channels due to key amino acid differences at the binding sites.

Our electrophysiology data support the idea that CBD primarily derives its effects in K_V_7.1 from binding at the S5–S6 site. This is because the activity of CBD was affected by mutations to S5–S6 site residues G272, A336, and F275. On the other hand, mutations of S338 from the S6–S5’–E1 site, which altered CBD response in K_V_7.1/KCNE1, had no effect on the CBD response in K_V_7.1. This supports our second proposal that the S6–S5’–E1 site can only exist in the presence of the KCNE1 subunit. Unlike K_V_7.1, however, mutations in both the S5–S6 and S6–S5’–E1 sites impacted the CBD response in K_V_7.1/KCNE1, in which both the G272C and A336G mutants altered CBD response. It can be argued that A336 is only two residues away from S338, which is important for the CBD response in K_V_7.1/KCNE1, but G272 is one helix away from the S6–S5’–E1 binding site. In contrast to WT K_V_7.1/KCNE1, the G272C mutant did not display a CBD-induced reduction in *G*_Max_ (however, a *V*_50_ response to CBD was observed for this mutant, which was not seen in WT K_V_7.1/KCNE1). In addition to S338, mutations to F53 and F57 of KCNE1 also influenced response to CBD, supporting the presence of the S6–S5’–E1 binding site. Therefore, we propose that in K_V_7.1/KCNE1, CBD primarily derives its response from binding at the S6–S5’–E1 site, but a portion of its response comes from CBD binding at the S5–S6 site, which exists regardless of the presence of the KCNE1 subunit.

MD simulations provided a means to explain this nuanced data for the S6–S5’–E1 binding site. We observed that the S6–S5’–E1 binding site constitutes a deeper pocket compared to the relatively shallow S5–S6 site (compare Fig. 4e and f). The implication of this is that CBD exited the S5–S6 site in a few simulation replicas but did not do so in any simulation replicas at the S6–S5’–E1 binding site (Fig. S9). The differences between the two pockets also seemed to manifest in the binding free energies of CBD. Specifically, CBD binding deep into the S6–S5’–E1 binding site conferred the most favourable binding free energy of the poses investigated. In essence, the presence of the KCNE1 subunit in K_V_7.1/KCNE1 creates a deep pocket for CBD to bind, which is not present in K_V_7.1 alone.

A striking observation from our electrophysiology experiments is that mutation of S338 and KCNE1 residues F53 and F57 to alanine comparably improved the potency of CBD in K_V_7.1/KCNE1. These residues formed favourable molecular interactions with CBD, with F53 having the additional role of forming a cover over the CBD molecule together with F335. Furthermore, we observe that KCNE1 residues F53 and F57 may stabilise binding site residues F270 and L266, respectively (Fig. S10), though it is unclear if this stabilisation persists when mutated to the smaller alanine residue. Evidently, if S338 and KCNE1 residues F53 and F57 play a role in forming favourable molecular or stabilising interactions, it is surprising that alanine mutations at these residues improve CBD potency in experiments. A plausible explanation is provided by the consistency of the potency shift, which suggests a shared trend whereby mutation to the smaller alanine residue favourably expands the space available within the S6–S5’–E1 binding site. In essence, we suspect that despite their favourable contacts, these residues may sterically constrain CBD within the pocket, and a more favourable/stable binding mode may be achieved by alleviating this strain.

Beyond forming part of the S6–S5’–E1 binding site, the KCNE1 subunit seemed to modulate the accessibility of the S5–S6 pocket. Even without CBD present, the KCNE1 subunit seemed to push the S6 helix, reducing the gap between the S5 and S6 helices. This gap, located between G272 and A336, is needed to allow CBD binding in K_V_7.1 by housing its limonene group. When CBD is bound in the S6–S5’–E1 site, this seemed to further push the S6 helix to the point where the S5–S6 binding site gap disappeared. These observations are consistent with our proposal that CBD in K_V_7.1/KCNE1 derives its effect primarily from binding to the S6–S5’–E1 site.

Based on sequence comparisons, the described S5–S6 binding site for CBD in K_V_7.1 is likely not present in neuronal K_V_7s, which have bulkier residues in place of G272 and a glycine in place of A336. However, this site appears to be part of a modulatory hub for other K_V_7.1 selective compounds, including the channel activators ML277 and R-L3. Structures of ML277 bound to *Xenopus* and human K_V_7.1 [13, 18] show that ML277 overlaps with the S5–S6 CBD site proposed here (see also Fig. S6), which aligns with our experimental data, suggesting that CBD and ML277 use the same site for binding. Mutations of residues in this pocket have been previously described to impact the effect of R-L3 and ML277 [41, 42] (see ref. [24] for a comparison of important residues). Although mutagenesis of specific residues lining the S5–S6 binding site in K_V_7.1 shows differences in how they impact the effect of ML277, R-L3, and CBD [18, 41, 42], the geometry of the space allotted by A336 and G272 appears to be critical for all three compounds: ML277 [41], R-L3 [42], and CBD (this study). Of note, both ML277 and R-L3 lose their effect with saturating levels of KCNE1 [43]. Given their overlapping binding sites with our S5–S6 CBD site, it is tempting to speculate that the reduced gap between the S5 and S6 helices induced by KCNE1 may contribute to this phenomenon.

On the other hand, the S6–S5’–E1 binding site for CBD in K_V_7.1/KCNE1 overlaps with the lower CBD molecule in the K_V_7.2 structure and with the retigabine binding site of neuronal K_V_7 channels. Although K_V_7.1 lacks a tryptophan at position 266 that is crucial to retigabine binding in K_V_7.2–K_V_7.5 [21, 44], mild retigabine-induced inhibition of K_V_7.1 and K_V_7.1/KCNE1 has been described [44, 45]. Based on the available literature, it is unclear whether retigabine mediates inhibiting effects on K_V_7.1 and K_V_7.1/KCNE1 via the same retigabine site as in neuronal K_V_7 subtypes. A superimposition of the CBD-bound K_V_7.2 structure suggests that L266 and F335 of K_V_7.1 can sterically hinder CBD binding in this site. As suggested above, KCNE1 co-assembly with K_V_7.1, apart from forming part of the pocket, seems to stabilise L266 and F335 in a conformation that is receptive to CBD binding. The implication of this is that compounds can be created to exploit the pocket created by the KCNE1 subunit to achieve K_V_7.1/E1 selectivity.

In the present work, we propose that the presence of the KCNE1 subunit contributes an alternative and preferred CBD binding site in K_V_7.1/KCNE1. A critical role of KCNE1 in forming binding sites has been previously described for mefenamic acid, DIDS, and adamantane compounds like AC-1 (also called JNJ303) [46, 47]. Our S6–S5’–E1 binding site is distinct from that of the channel activators mefenamic acid and DIDS, which are found at the extracellular interface between K_V_7.1 and KCNE1 (near KCNE1 residue 41) [46]. In contrast, our S6–S5’–E1 binding site is in the same region as that of AC-1; however, different residues are important for their effects ([47] and the present study).

It is also interesting to note that regions encompassing both the S5–S6 site and the S6–S5’–E1 site can harbour compounds inducing either activating or inhibiting K_V_7 channel effects. For instance, although CBD, ML277, and R-L3 likely have overlapping binding sites in K_V_7.1, CBD inhibits K_V_7.1, whereas ML277 and R-L3 activate the channel. We note that the ML277 binding mode spans far beyond the S5–S6 site here and interacts with the S4﻿–S5 linker as well as the S5’ and S6’ helices of the adjacent subunit [17, 18]. For R-L3, the compound has been proposed to extend beyond the S5–S6 site by interacting with the S4 helix of the channel [48]. It may be that interactions with a single K_V_7.1 subunit underlie the inhibitory effect of CBD. Further work is needed to understand whether such differences contribute to an activating *versus* inhibiting effect. Similarly, although the retigabine site in K_V_7.2, overlapping with our S6–S5’–E1 site, is mostly characterised for channel activators, inhibitors like Ebio3 and ML252 use the same site [15, 49]. Altogether, growing evidence shows that both activators and inhibitors can utilise pockets like the S5–S6 and S6–S5’–E1 sites described here. Further studies are required to understand the mechanistic basis for the diverse effects. Moreover, future studies are needed to determine how CBD inhibits the channels from the S5–S6 and S6–S5’–E1 sites. Such studies could potentially utilize Cryo-EM and MD simulations starting from different pore state conformations to explore potential mechanisms underlying CBD-induced channel inhibition. For instance, it would be interesting to see if CBD impacts electromechanical coupling, as has been discussed for other compounds using the S5–S6 site and the retigabine site in K_V_7 channels (summarized in our previous study [24]), or induces similar “squeezing” of the S6 helix as has been proposed for the non-blocking inhibitory effect of Ebio3 in K_V_7.2 [15].

In this study, we used Chai-1 as a starting point to identify potential CBD binding sites. This presents a shift in computational binding site determination studies, where docking and MD investigations are typically guided by initial site-directed mutagenesis findings, as they are unsuitable for exploring all possible binding sites in large proteins. Although the Chai-1 models showed low prediction confidence (ipTM ≤ 40), generating 100 CBD poses in K_V_7.1 and K_V_7.1/KCNE1 broadened the sampling and demonstrated a trend where top-scoring poses favoured specific binding sites. Site-directed mutagenesis validated these sites, after which induced-fit docking generated tighter CBD binding poses, and MD simulations examined their behaviour under physiologically relevant, lipid-bilayer conditions. Overall, this workflow demonstrates how AI-based prediction, combined with docking and MD, can efficiently map and refine ligand-binding sites.

One limitation of this study is that at the beginning of this project, no structure for K_V_7.1 bound to KCNE1 had been resolved. However, with the recently deposited structures by Cui et al. [19], we have confirmed that our K_V_7.1/KCNE1 model closely matches the closed state K_V_7.1/KCNE1 structure (Fig. S1). Another limitation is that we could not answer why some residues that favourably interacted with CBD did not alter the response when mutated (F335 and F339 in K_V_7.1 and L266 in K_V_7.1/KCNE1). It is possible that the presented binding modes are stable in spite of mutations to these residues or that more stable binding modes are possible, where CBD shifts slightly to reduce interactions with these residues. Another limitation is the discrepancy between the present study and our previous work [24] regarding the impact of the F335A mutation on CBD effects. Our previous data showed that the F335A mutation in K_V_7.1 alone reduced the inhibiting effect of 30 µM CBD on *G*_Max_, from −71%  ±  5% for WT to −45%  ±  4% for F335A [24]. The reduced effect on *G*_Max_ for this mutant was not seen in the present study (Fig. 2a). We believe that the discrepancy is caused by differential loss of CBD in different perfusion systems, from CBD binding to plastic surfaces, as has been pointed out by several previous studies [23, 24, 50]. In our previous study, experiments on K_V_7.1 were performed on two electrophysiology setups with minor differences in the perfusion system. In the present study, in which all experiments were performed using the same electrophysiology setup, no difference in the CBD response was observed between K_V_7.1 WT and F335A (Fig. 2a; Fig. S12). Thus, we acknowledge that some CBD may still be lost in our perfusion system; however, as all experiments were carried out using the same setup in this work, we have maintained internal consistency.

In conclusion, we have provided evidence that CBD inhibits K_V_7.1 and K_V_7.1/KCNE1 through two distinct binding pockets. We envision that detailed characterisations of K_V_7.1 and K_V_7.1/KCNE1 binding sites for channel inhibitors, like ours for CBD, provide insights into how to avoid inhibiting adverse effects on cardiac K_V_7 subtypes. As CBD is being increasingly consumed, insights into potentially harmful effects are relevant and may inspire further studies in more complex experimental models to evaluate potential risks on cardiac repolarisation. Additionally, detailed characterisation of KCNE1’s role in binding site formation enables rational design of compounds that selectively activate K_V_7.1/KCNE1. Drugs may be designed to better target the S6–S5’–E1 binding site with the aim of treating cardiac arrhythmias related to the K_V_7.1/KCNE1 channel.

## Supplementary information

Supplementary information

## Data Availability

All data underlying conclusions from electrophysiology experiments are provided in the main figures or Supplementary Information. Molecular dynamics trajectories are available on Zenodo with DOI 10.5281/zenodo.17778963. Supplementary information is available at the website of Acta Pharmacologica Sinica.
