# Supplementary material for: Cannabidiol inhibits both human KV7.1 and KV7.1/KCNE1 channels through distinct sites
Source: Acta Pharmacol Sin. 2026 Mar 3;47(7):1825–39. doi: 10.1038/s41401-025-01742-0 (PMC13279787; doi:10.1038/s41401-025-01742-0)
Supplement: Supplementary file 1 — Supplementary information [file 41401_2025_1742_MOESM1_ESM.pdf]

Supplementary information

Cannabidiol inhibits both human $K_V7.1$ and $K_V7.1$/KCNE1 channels through distinct sites

Kusay AS, Pökl M, Hiniesto-Iñigo I, Sridhar A, Delemotte L, Liin SI

SI Table 1: Summary of the intrinsic properties of WT $K_V7.1$ and $K_V7.1$ mutants under control conditions and indicated effects of 30 µM of CBD.

	Control					30 µM CBD		
	V_{50}	SEM	s (mV)	SEM	n	Δs (mV)	SEM	N
WT	+1.4	0.8	11.8	0.2	16	−1.6	0.5	16
L266W	+5.5	0.9	11.6	0.7	7	−1.9	0.4	5
G272C	+4.1	0.3	8.7	0.2	10	0.0	0.2	6
F275A	+21.6	1.9	12.5	0.3	10	1.6	0.6	10
S276A	+0.2	0.8	11.2	0.4	7	−0.8	0.3	7
F335A	−19.1	3.3	14.2	0.5	5	−1.5	0.5	5
A336G	+7.7	0.9	11.3	0.3	13	−0.4	0.4	7
S338A	+5.5	3.4	18.7	1.1	7	−1.5	1.4	7
F339A	−1.1	1.0	13.9	0.3	7	−3.0	0.5	7

V_{50} denotes voltage generating half maximal conductance. s denotes slope of curve. Δs denotes CBD-induced change in slope. n denotes sample sizes (number of oocytes).

SI Table 2: Summary of the intrinsic properties of WT $K_V7.1$/KCNE1 and $K_V7.1$/KCNE1 mutants under control conditions and indicated effects of 30 μM of CBD.

	Control					30 μM CBD		
	V_{50}	SEM	s (mV)	SEM	n	Δs (mV)	SEM	n
**KCNE1 WT**								
$K_V7.1$ WT	+24.4	2.6	18.1	0.6	7	−2.5	0.6	7
$K_V7.1$ L266W	+17.2	2.6	25.9	0.6	6	−4.7	1.0	6
$K_V7.1$ G272C	+25.1	1.7	19.9	0.7	9	−2.8	0.7	9
$K_V7.1$ A336G	+22.6	1.6	21.4	1.0	6	1.0	0.7	6
$K_V7.1$ S338A	+53.7	2.9	15.6	1.2	6	−4.0	1.0	6
$K_V7.1$ S338W	+46.6	3.9	20.3	0.7	5	4.8	0.5	5
**KCNE1 F53A**								
$K_V7.1$ WT	+47.1	2.0	17.7	1.1	5	−2.8	0.6	5
**KCNE1 F57A**								
$K_V7.1$ WT	+32.2	1.9	13.5	0.6	12	−0.7	0.4	7

V_{50} denotes voltage generating half maximal conductance. s denotes slope of curve. Δs denotes CBD-induced change in slope. n denotes sample sizes (number of oocytes).

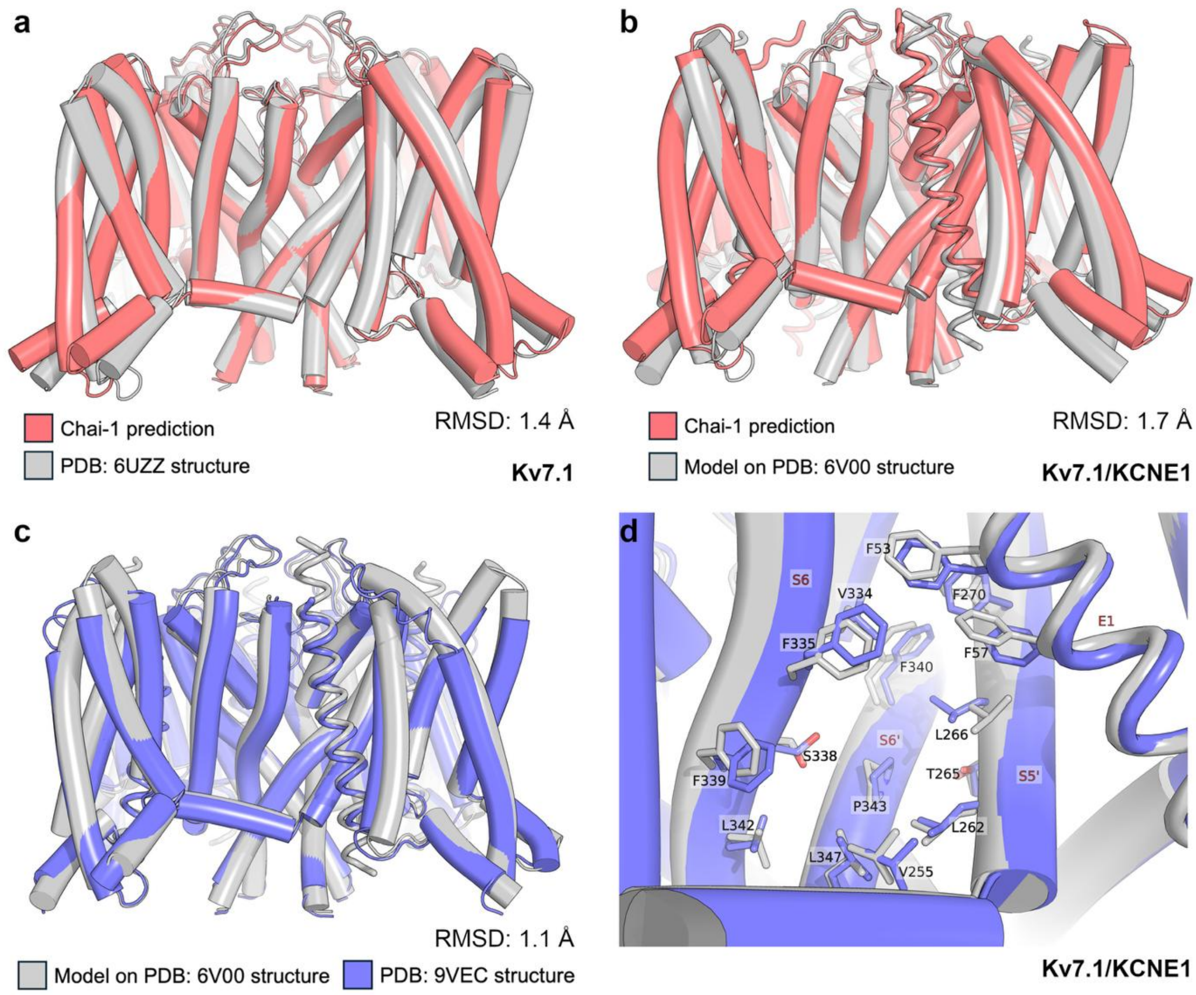

Figure S1: Comparison between K$_V$7.1 structure and K$_V$7.1/KCNE1 model/structure and their respective Chai-1 predictions. The K$_V$7.1/KCNE1 model was created based on the K$_V$7.1/KCNE3 structure (PDB: 6V00)[1]. **a** The Chai-1 predicted K$_V$7.1 is superimposed on the K$_V$7.1 structure (PDB: 6UZZ) (RMSD: 1.4 Å). **b** The Chai-1 predicted K$_V$7.1/KCNE1 is superimposed on the K$_V$7.1/KCNE1 model (RMSD: 1.7 Å). **c**−**d** The K$_V$7.1/KCNE1 model is superimposed on the closed state K$_V$7.1/KCNE1 structure (PDB: 9VEC)[2] (RMSD: 1.1 Å), only the transmembrane section of the K$_V$7.1/KCNE1 structure is displayed (**c**), a close up of the S6–S5'–E1 binding site is displayed (**d**). In both K$_V$7.1 and K$_V$7.1/KCNE1, the pore domain and voltage sensing domain positions were overlapped well. The low overall RMSD indicates that Chai-1 predictions and K$_V$7.1/KCNE1 model are structurally similar to the K$_V$7.1 and K$_V$7.1/KCNE1 structures. Furthermore, the K$_V$7.1/KCNE1 model used in this study closely captures the S6–S5'–E1 site in the recently published closed state apo K$_V$7.1/KCNE1 structure.

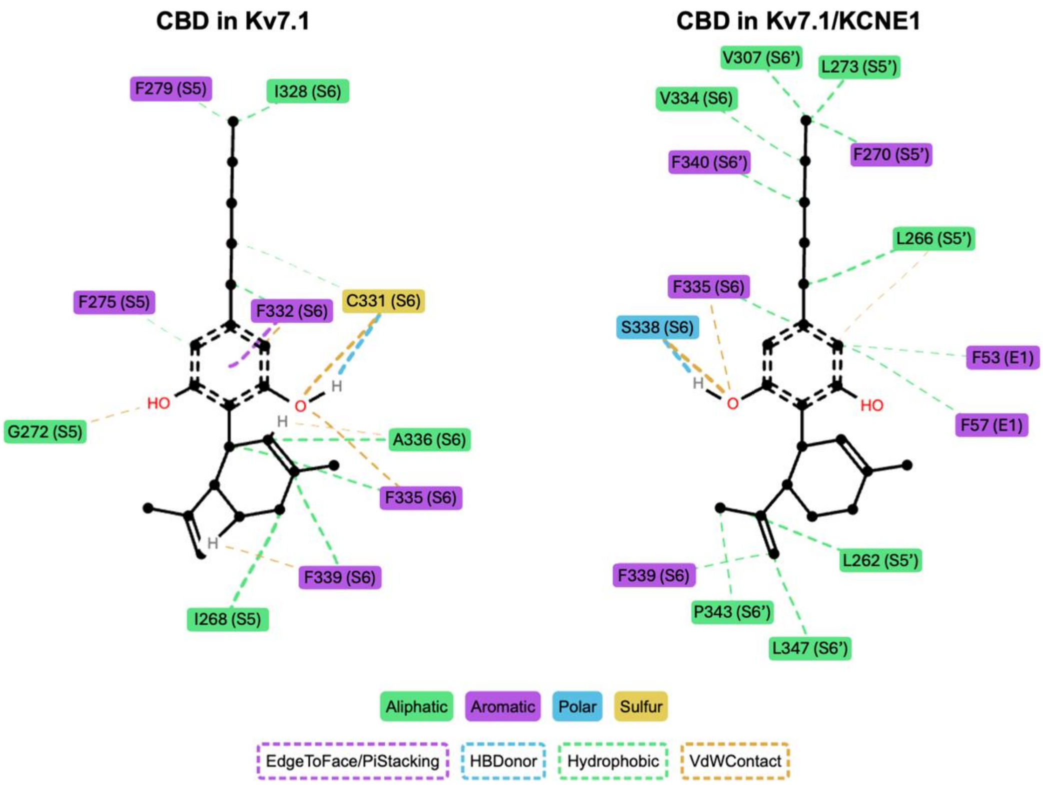

Figure S2: Prominent molecular interactions observed in the most populated binding pose clusters. Molecular interactions between CBD and protein residues were calculated using the ProLIF tool[3]. Only interactions present for >33% of cluster frames are shown, thicker lines represent more prominent interactions. The H-bond interactions were counted if the distance between acceptor/donor heavy atoms was <3.5 Å and if the angle between the donor heavy atom, donor hydrogen, and acceptor heavy atom was between 130–180˚. The edge-to-face π-stacking interactions were counted if the distance between the ring centroids was <6.5 Å and the angle between the two ring planes was between 50–90˚. The hydrophobic interactions were counted if the distance between non-polar atoms was <4.5 Å. The vdW contacts were counted if the distance between any two atoms was less than the sum of their vdW radii.

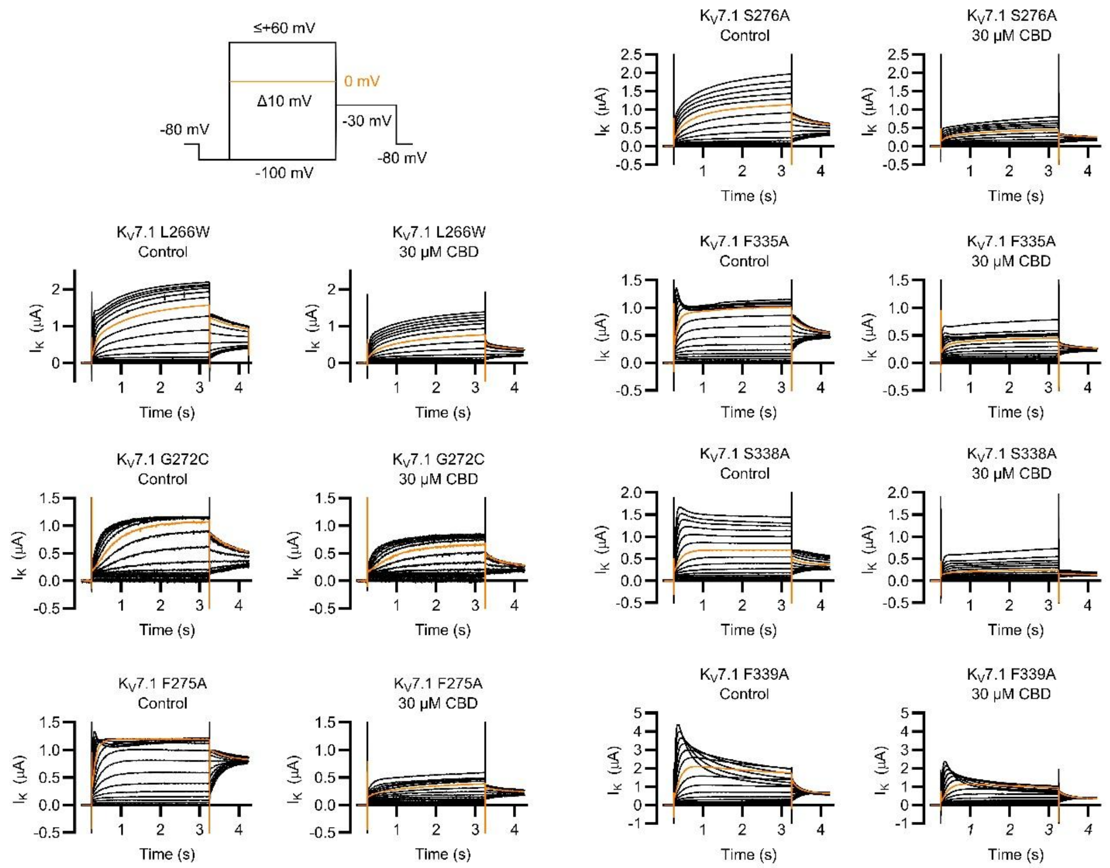

Figure S3: Representative traces of K_V7.1 WT and mutants under control conditions and in the presence of 30 µM CBD. Inset shows voltage protocol. Orange sweep represents the current generated by a test pulse to 0 mV. Maximum voltage used depended on saturation of tail currents but did not exceed +60 mV.

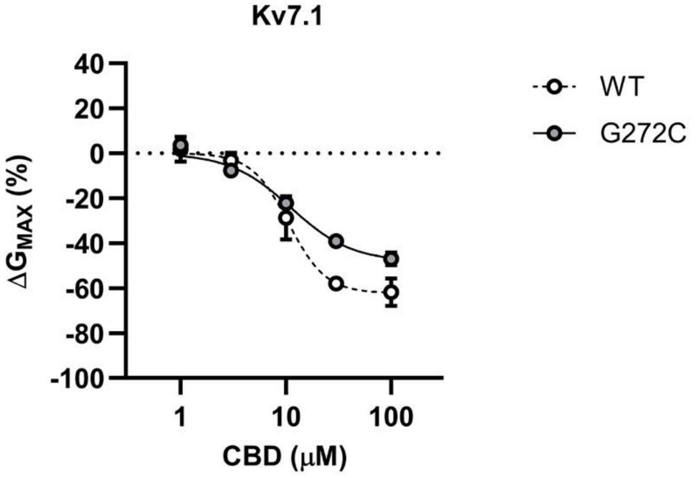

Figure S4: Concentration-response relationship for CBD effect on G_{Max} of $K_V7.1$ containing G272C. Concentration-response relationship for CBD effect on G_{Max} of $K_V7.1$ containing G272C (filled symbols). Data for WT $K_V7.1$ is included for comparison (dotted line and open symbols). Data displayed as mean ± SEM. n = 5−6 oocytes. Best fits: $K_V7.1$ WT: E_{Max} (i.e. maximal reduction in G_{Max} induced by CBD) = −62%; IC_{50} = 10.6 μM (95% CI: 7.6−15.4, R^2= 0,8343), G272C: E_{Max} = −48%; IC_{50} = 11.1 μM (95% CI: 8.0−17.1, R^2= 0,9278). Note that the IC_{50} for WT was higher than reported in Pökl *et al.*[4], likely due to slight differences in experimental perfusion systems (see Discussion).

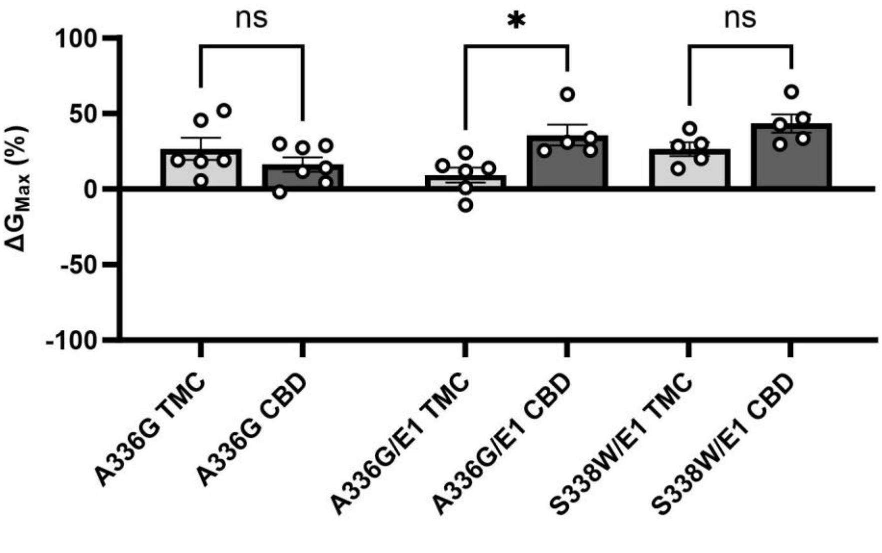

Figure S5: Comparison of time-dependent and CBD-induced change in G_{Max} for mutants with non-linear current run-up. Mean change in G_{Max} for indicated mutants in K_V7.1 alone or K_V7.1/KCNE1 (indicated by 'E1') induced by time (denoted 'TMC', no CBD added) or 30 µM CBD (denoted 'CBD', same data as in Figure 2a and 3a) in *Xenopus laevis* oocytes. n = 5−7 oocytes, with individual data points shown. Statistics denote Welch's ANOVA followed by Dunnett's test to compare indicated pairs, with ns > 0.05 and *P<0.05.

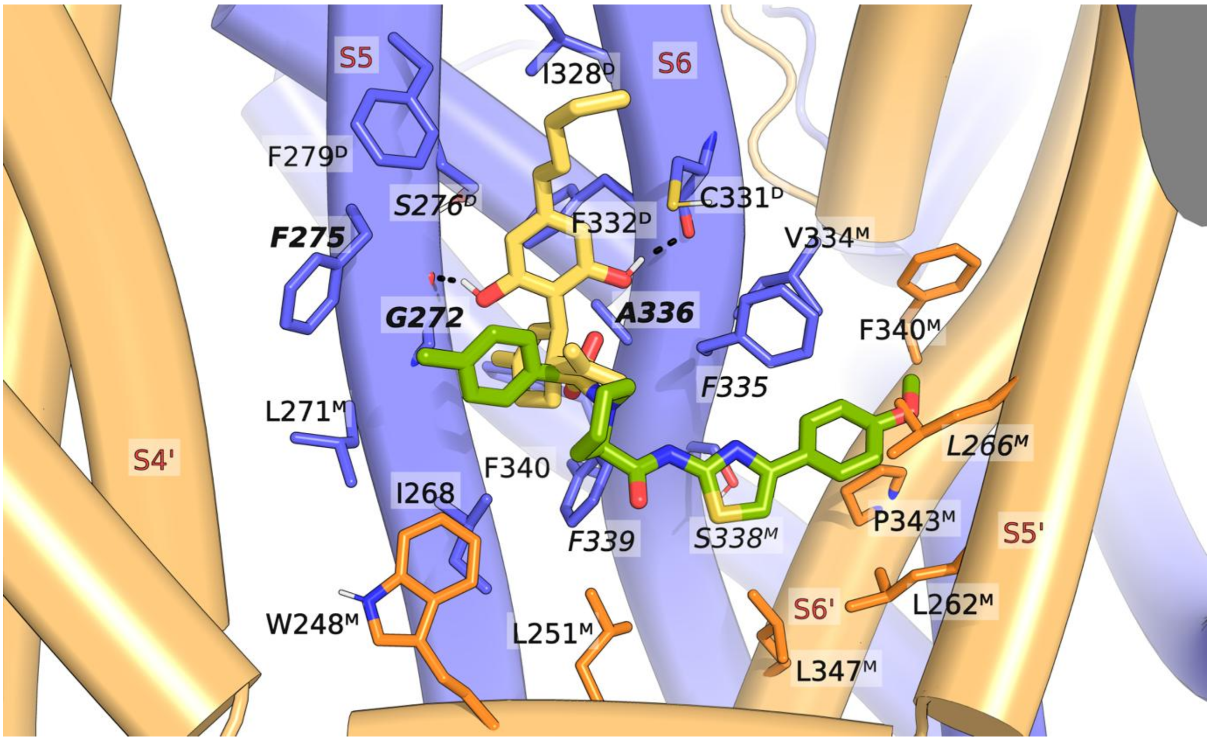

Figure S6: The docked pose of CBD in K_V7.1 is only possible in the absence of ML277. Top scoring docking pose of CBD (yellow) into the K_V7.1 structure (PDB: 6UZZ)[1], the ML277-bound K_V7.1 structure (PDB: 7XNL)[5] is overlaid on the CBD docked K_V7.1 structure, only ML277 (green) is shown to highlight the overlap with CBD. Residues adjacent to CBD or ML277 are shown, a "D" and "M" superscript are used to denote residues within 4 Å of CBD or ML277, respectively; residues within 4 Å of both do not have a superscript. Residues that were tested for CBD using site-directed mutagenesis are italicised, residues with a significant difference compared to WT are bolded as well. The right subunit is coloured orange; all other subunits are coloured in purple-blue.

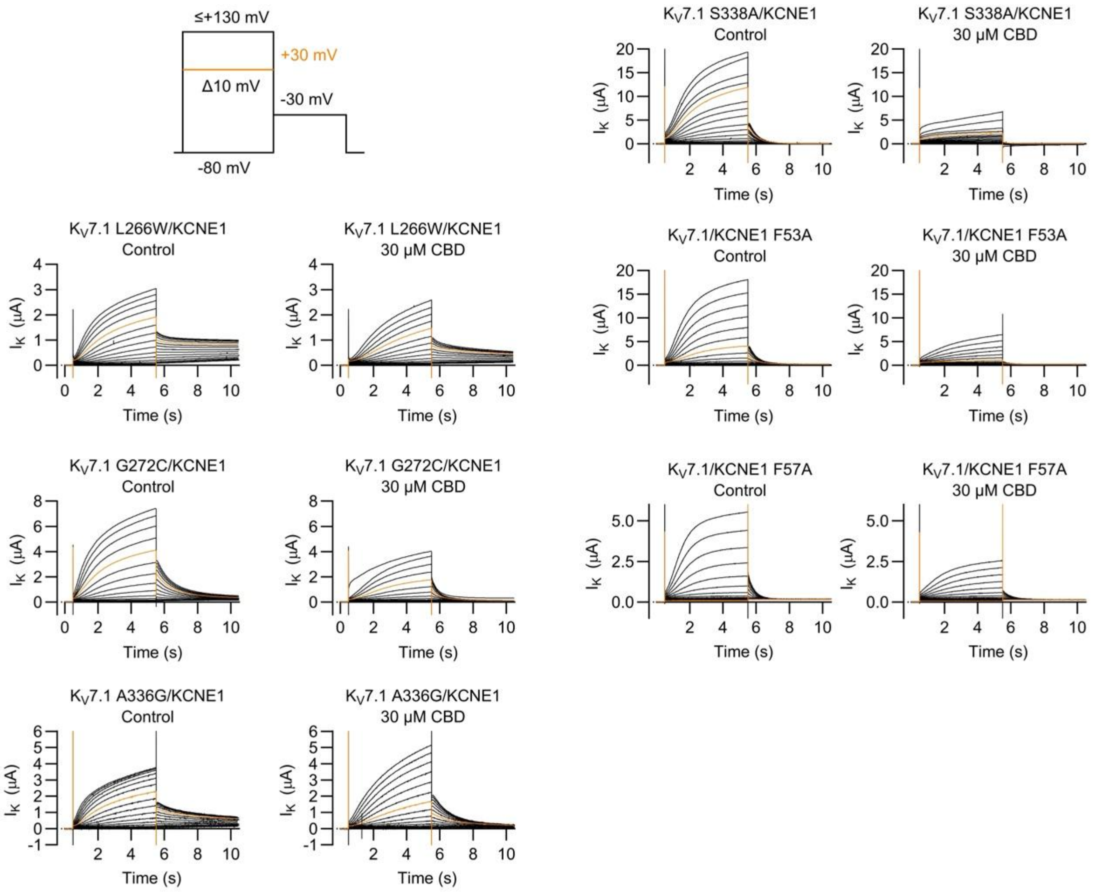

Figure S7: Representative traces of K_V7.1/KCNE1 WT and mutants under control conditions and in the presence of 30 µM CBD. Inset shows voltage protocol. Orange sweep represents the current generated by a test pulse to +30 mV. Maximum voltage used depended on saturation of tail currents but did not exceed +130 mV.

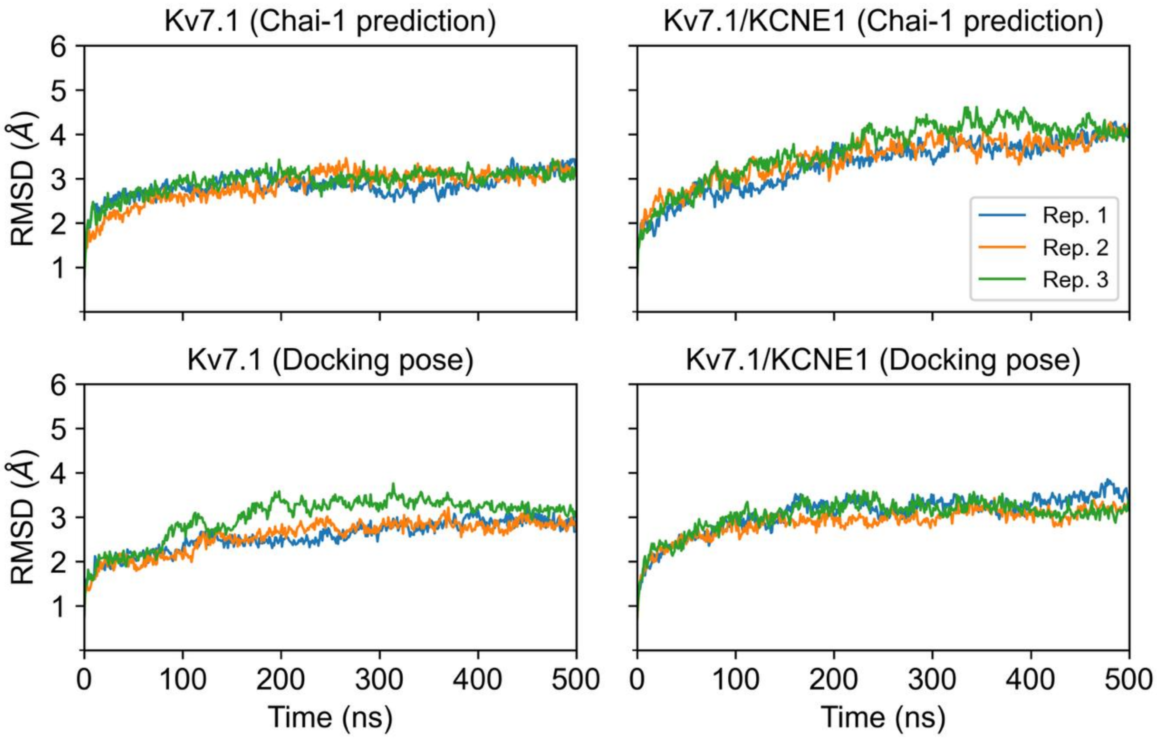

Figure S8: RMSD of the CBD bound K$_V$7.1 and K$_V$7.1/KCNE1 from the Chai-1 prediction and from docking poses. RMSD was calculated for the protein Cα atoms every 1 ns, following superimposition on the initiation conformation. Blue, orange and green represent, replicas 1, 2 and 3, respectively.

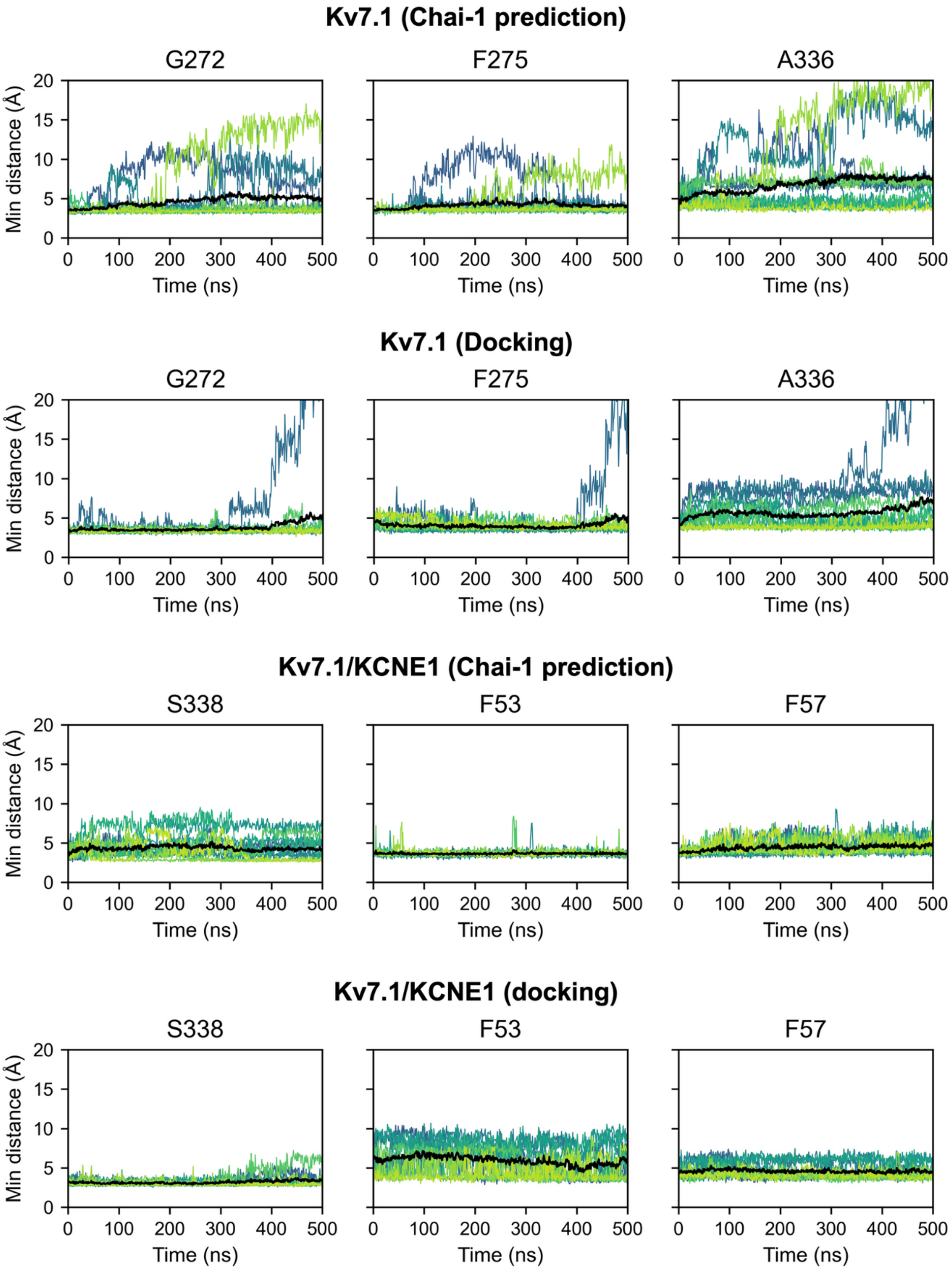

Figure S9 Minimum distance between CBD and key binding site residues in K_V7.1 and K_V7.1/KCNE1. n = 12. Plots coloured based on a purple to yellow colour spectrum represent 3 simulation replicas x 4 binding sites, the average is shown as a black line.

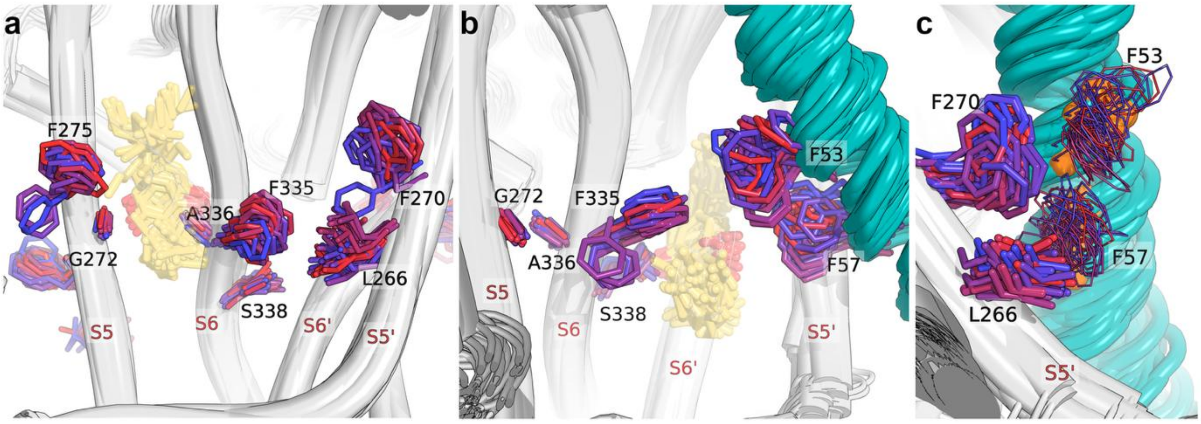

Figure S10: Spectrum of cluster frames, showing distribution of residue conformations. a–c CBD in KV7.1 (**a**), CBD in KV7.1/KCNE1 (**b**) and a panel highlighting how to KCNE1 residues F53 and F57 stabilise KV7.1 residues F270 and L266 (**c**), respectively. Given that F53 primarily interacted through its backbone, it is likely that the stabilising effect will persist even after mutation to the smaller alanine sidechain. For F57 however, it interacted primarily through its sidechain, so it is unclear if this stabilising effect can remain after mutation to alanine. All frames are superimposed on the initial protein conformation based on Cα atom positions in the S5 and S6 helices of all chains. A blue to red colour spectrum is used to illustrate the conformations of KV7.1 residues L266, F270, G272, F275, F335, A336 and S338 and KCNE1 residues F53 and F57 from 30 frames sampled uniformly from the simulation clusters. CBD is coloured in yellow and shown in a faded representation, KCNE1 is coloured in teal, other subunits are coloured white. The orange spheres in panel **c** represent the Cα atoms of F53 and F57.

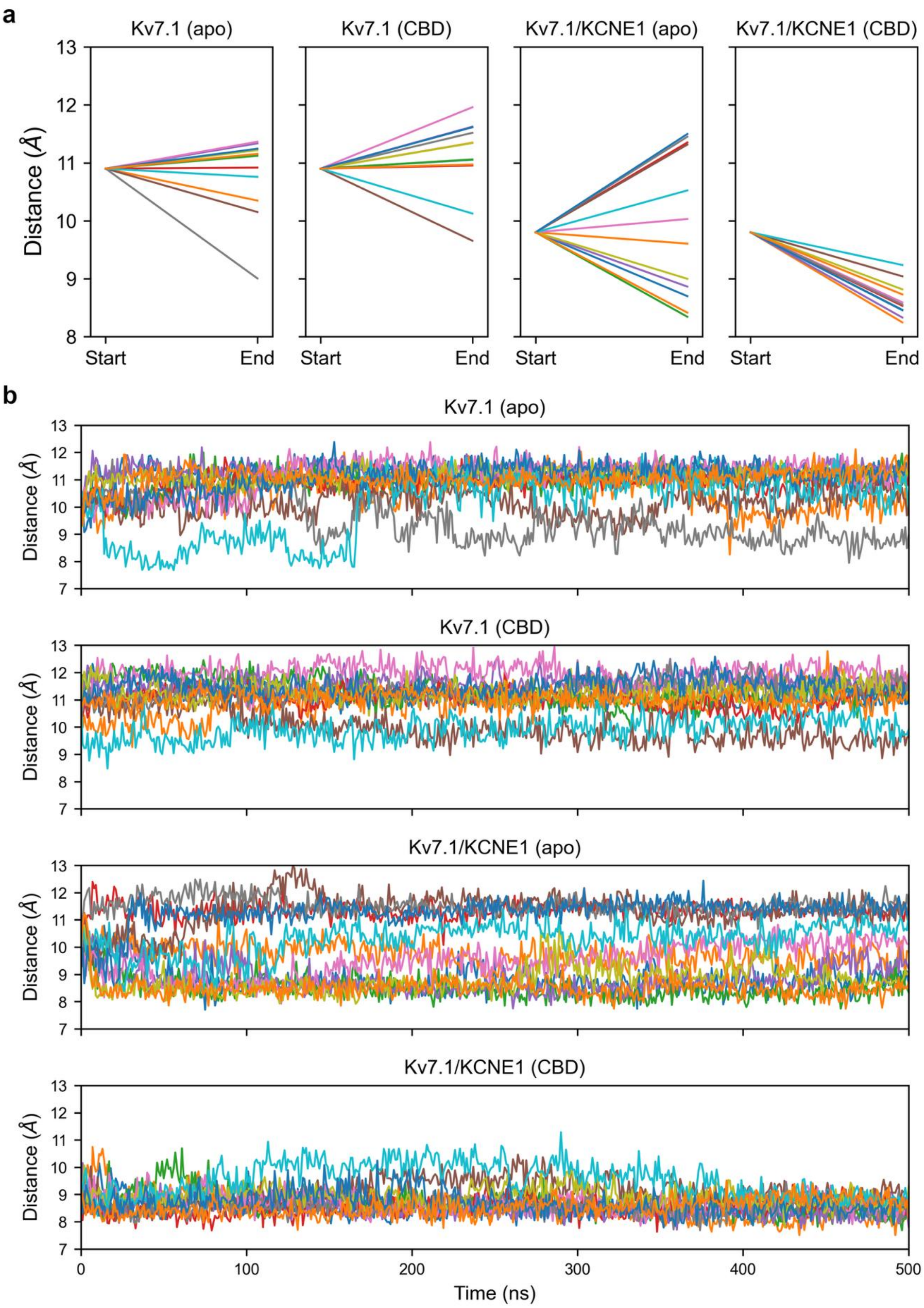

Figure S11: Distance between the S5 and S6 helices in K_V7.1 and K_V7.1/KCNE1, in the presence or absence of CBD. **a** Plot of distances at the start (the S5–S6 helical distance from the initial docked poses of CBD in K_V7.1 and K_V7.1/KCNE1) and the end of the simulation. **a** Plot of distances over time. We note that the "Start" distance in panel **a** corresponds with the initial protein conformation whereas at time = 0 ns in **b**, the initial distance reflects the configuration following the equilibration protocol. n = 12 reflects, 3 simulation replicas x 4 binding sites.

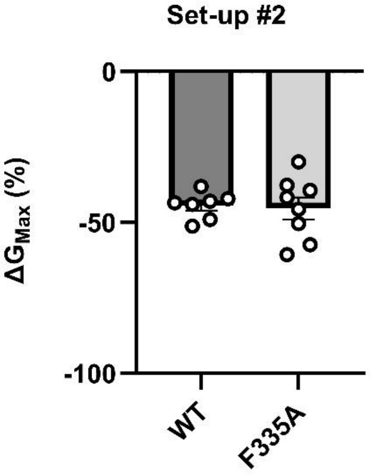

Figure S12: Comparison of CBD effect on G_{Max} of K_V7.1 WT and K_V7.1 F335A in the experimental setup used in Pökl et al.[4]. Data shown as mean ± SEM. Note that 30 μM CBD has a slightly larger effect on K_V7.1 WT in this experimental setup compared to the one used in the present study (Figure 2a), likely due to minor differences in the perfusion system and recording chamber. However, when the F335A mutant is tested in the same setup, the CBD response does not differ from that of the WT channel.

References

1 Sun J, MacKinnon R. Structural Basis of Human KCNQ1 Modulation and Gating. Cell 2020; 180: 340-7.e9.

2 Cui C, Zhao L, Kermani AA, Du S, Pipatpolkai T, Jiang M*, et al*. Mechanisms of KCNQ1 gating modulation by KCNE1/3 for cell-specific function. Cell Res 2025.

3 Bouysset C, Fiorucci S. ProLIF: a library to encode molecular interactions as fingerprints. Journal of Cheminformatics 2021; 13: 72.

4 Pökl M, Sridhar A, Frampton DJA, Linhart VA, Delemotte L, Liin SI. Subtype-specific modulation of human Kv7 channels by the anticonvulsant cannabidiol through a lipid-exposed pore-domain site. British Journal of Pharmacology 2023; 180: 2956-72.

5 Ma D, Zhong L, Yan Z, Yao J, Zhang Y, Ye F*, et al*. Structural mechanisms for the activation of human cardiac KCNQ1 channel by electro-mechanical coupling enhancers. Proc Natl Acad Sci U S A 2022; 119: e2207067119.
